# Chloroplasts as ingredients for food: a review

**DOI:** 10.1039/d5fo03797b

**Published:** 2026-06-04

**Authors:** David A. Gray, Poramat Sutcharit, Jutarat Wattanakul, Mohamed A. Gedi, Ruth Price, Ardeshir Farmanfarmaian, Randa Darwish, Syamila Mansor, Chao Chi, Malgorzata Walczak, Rhianna Briars, Joshua E. S. J. Reid, Vincenzo Di Bari, Joanne Gould, Molly Muleya, Robert Rintoul, Lorna Mcausland, Moulay Sahaka, Frédéric Carrière

**Affiliations:** a Division of Food, Nutrition and Dietetics, School of Biosciences, University of Nottingham Sutton Bonington Campus Loughborough Leicestershire LE12 5RD UK david.gray@nottingham.ac.uk +44(0) 115 951 6147; b Department of Food Sciences and Technology, Faculty of Home Economics Technology, Rajamangala University of Technology Krungthep Bangkok 10120 Thailand; c Food Biotechnology, Faculty of Science and Technology, Universiti Sains Islam Malaysia Nilai 71800 Malaysia; d Division of Plant and Crop Sciences, School of Biosciences, University of Nottingham Sutton Bonington LE12 5RD UK; e Aix Marseille Univ, CNRS, UMR7281 Bioénergétique et Ingénierie des Protéines 31 chemin Joseph Aiguier F-13402 Marseille cedex 20 France

## Abstract

The chloroplast is best known for its role in photosynthesis, the process by which sunlight energy is converted into chemical energy in the form of sugar. Research conducted at the University of Nottingham (UK) over several years has revealed the nutritional composition of the chloroplast and the physical properties of its multicomponent membrane. These attributes qualify this globally ubiquitous organelle to be a natural ingredient in food products and to be an option to tackle specific nutrient deficiencies across the globe. Detailed studies of the biochemistry of photosynthesis require pure preparations of enzymatically active chloroplasts, requiring various stages of lab-scale extraction, separation and purification. Such an approach may be commercially viable for pharmaceutical applications, but not for food ingredients. It is against this background that we have developed a simple process to recover a chloroplast-rich fraction (CRF) that could be used in food products. After introducing the reader to the nature of chloroplasts, this review: presents the method we have developed to extract and stabilise a chloroplast-rich fraction; summarises the composition of chloroplast-rich fractions gleaned from spinach leaves and from the postharvest field residue ‘pea vine haulm’ (PVH); explores the impact of drying methods on the physical nature and composition of the CRF material; establishes the impact of heat-treatment on the quality of CRF material; presents the evidence for extensive galactolipid digestion processes in the human gastrointestinal (GI) tract; investigates the release of nutrients from CRF material during digestion; briefly covers the surface-active properties of the multicomponent membrane system/thylakoids/chloroplast membrane material (CMM).

## Abstract 2

The chloroplast is best known for its role in photosynthesis, the process by which sunlight energy is converted into chemical energy in the form of sugar. This image is created using Biorender.

## Graphical abstract

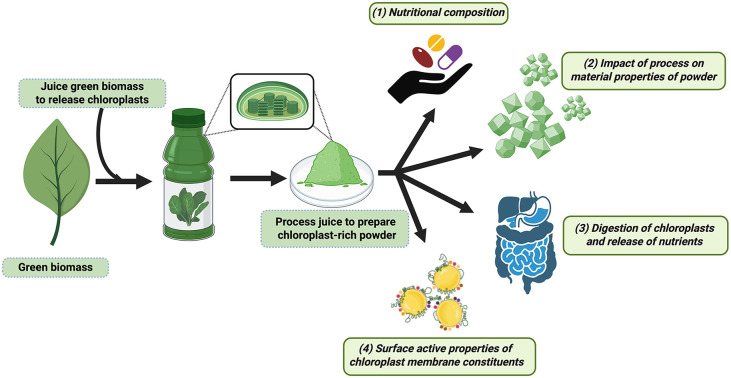

## 1. Chloroplasts – a very brief introduction

**Fig. 1:**
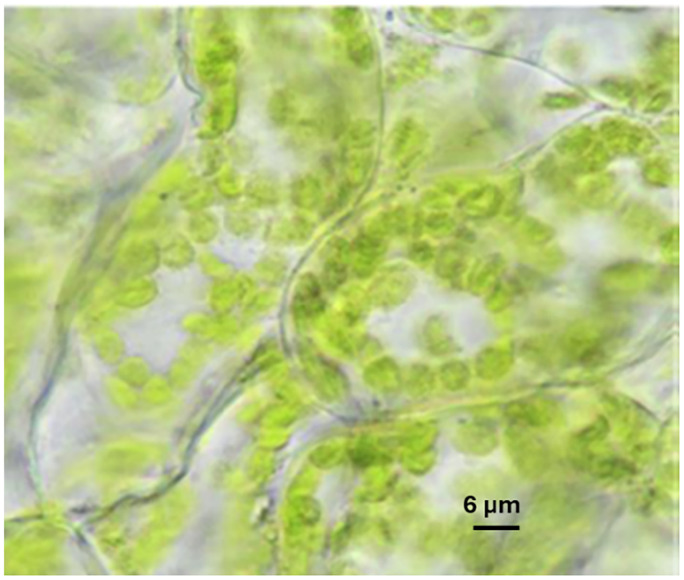
Light micrograph of a cross-section of spinach leaf. The green structures are chloroplasts. The image was taken by Sucharit, (2022).^81^

Chloroplasts (Fig. 1) are membrane-bound sub-cellular organelles 3 to 10 µm in diameter found in plants and algae; they are responsible for converting sunlight energy into chemical energy (the light-dependent reaction of photosynthesis), and harnessing this energy to fix carbon dioxide through the action of ribulose-1,5-bisphosphate carboxylase/oxygenase (RuBisCO) to make sugar (the dark reaction or Calvin–Benson cycle, also known as the Calvin cycle).^1^

Due to their ubiquity in the biosphere, and their numerous stacks of membranes, they contain the world's most abundant lipid (galactolipids, which are composed predominantly of the omega-3 fatty acid α-linolenic acid) and protein (ribulose-1,5-bisphosphate carboxylase/oxygenase, RuBisCO found in the aqueous stromal region).

**Fig. 2:**
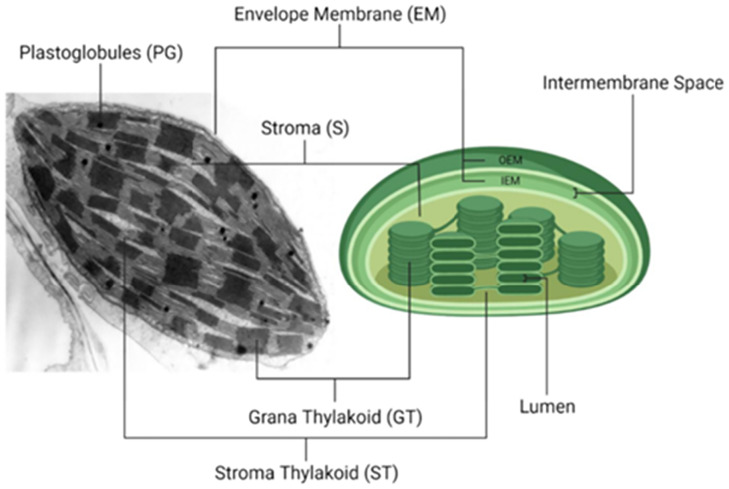
Overview of the chloroplast structure as a transmission electron micrograph (TEM) (left) and as an illustration (right). Some visible microstructures of the chloroplast are the envelope membranes (EM) (inner-envelope [IEM] and outer envelope membranes (EM) (inner-envelope [IEM] and outer envelope membranes [OEM]), stroma (S), grana thylakoid (GT), stroma thylakoid (ST; also refer to as lamellae) and plastoglobuli (PG). The space between the thylakoid membranes is referred to as the lumen, and the space between the IEM and OEM is referred to as the intermembrane space. The TEM micrograph was taken from Shutova, 2007.^2^

**Fig. 3:**
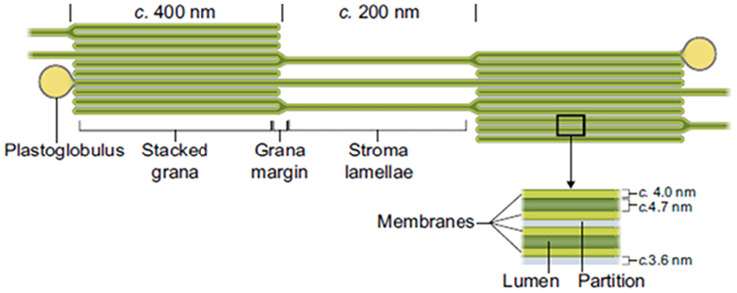
In-scale model of a thylakoid membrane cross section. The grana diameter can vary between 350 and 600 nm. The model reveals the strict stacking of membranes in the grana area. The diameter of plastoglobuli is typically between 45 and 60 nm but can vary under certain conditions. Image taken from Kirchhoff, 2019.^3^

The ultrastructure of a chloroplast is complex but can be described as a spherical or oblate organelle laced with stacked thylakoid membranes (80% of chloroplast lipid) throughout its interior and surrounded by an outer and inner membrane (20% of chloroplast lipid) (Fig. 2). The term ‘thylakoid’, from the Greek word ‘thylakos’ meaning ‘sac’, was first introduced by Menke^4^ following the first high-resolution micrograph of thylakoid grana consisting of multiple 7–9 nm thick membrane structures:^5^ see Fig. 3 for an annotated schematic of thylakoid membranes with dimensions. Proteins (50–60% DWt) and lipids (25–30% DWt) make up the majority of the chloroplast's components and they are arrayed in an elegant fashion to efficiently harvest light energy and to synthesise sugar molecules.

**Fig. 4:**
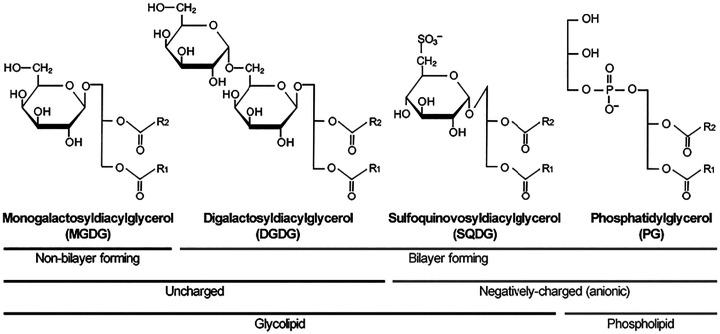
Structure and characteristic of glycerolipids in the thylakoid membrane. Major glycerolipids in the thylakoid membrane of plants and cyanobacteria can be classified into non-bilayer-forming (MGDG) and bilayer-forming lipids (DGDG, SQDG and PG), uncharged (MGDG and DGDG) and negatively-charged lipids (SQDG and PG), and glycolipids (MGDG, DGDG, SQDG) and phospholipids (PG). *R*_1_ and *R*_2_ denote hydrocarbon chains of fatty acids, which is predominantly α-linolenic acid. Taken from Kobaysahi, 2016.^7^

Recent research using cryo-EM, cryo-ET and cryo-AFM (electron microscopy, electron tomography, and atomic force microscopy respectively), in combination with the creation of plant and algal genetic mutants, has shed light on the ultrastructure of the thylakoid membrane, the major constituent of chloroplasts that forms a 3-D continuous network enclosing an aqueous region called the lumen. These ordered arrays of stacked bilayer membranes not only provide an anchor for photosynthetic complexes rich in transmembrane proteins, but act as insulators preventing passive diffusion of the proton gradient that is generated during the light dependent reaction of photosynthesis where solar energy is harnessed to split water into oxygen and protons, leading, through a series of redox reactions, to the generation of chemical energy (NADH and ATP). There are four distinct protein complexes involved in the light-dependent reactions of photosynthesis: photosystem II (PSII), cytochrome b_6_f (cyt b_6_f), photosystem I (PSI), and F-ATPase; these complexes occupy 70% of the thylakoid membrane. Thylakoid membranes are composed of the following glycerolipids MGDG, DGDG, SQDG and PG, each present at 50, 30, 10, and 10 mol% respectively^6^ (see Fig. 4).

**Fig. 5:**
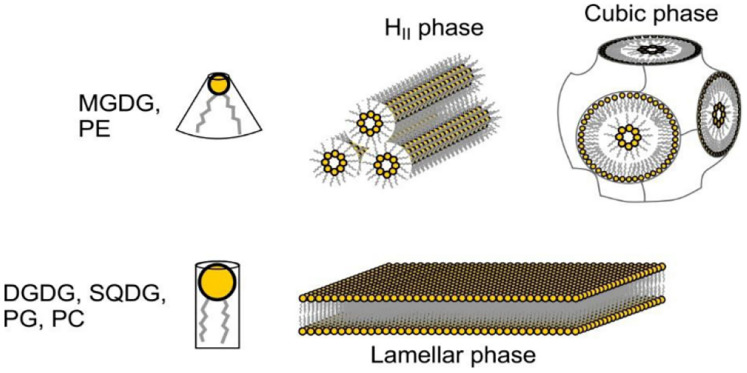
Schematic representation of thylakoid membrane lipids and some additional phospholipids (PE and PC), and their spontaneously formed lipid phases in the presence of water. The main thylakoid membrane lipid, MGDG (monogalactosyldiacylglycerol), and PE (phosphatidylethanolamine), because of their conical shapes, prefer to form inverted hexagonal (H_II_) phase or cubic phase (which also shows isotropic arrays of lipid molecules) – they are thus called non-bilayer or non-lamella forming lipids. The lipid molecules possessing cylindrical shapes, DGDG (digalactosyldiacylglycerol), SQDG (sulfoquinovosyldiacylglycerol), PG (phosphatidylglycerol) and PC (phosphatidylcholine) form lamellar phase – and are called lamella-forming or bilayer lipids. (Taken from Garab *et al.*, 2016 ^10^ and represented in Wilhelm *et al.*, 2020.^11^)

**Fig. 6:**
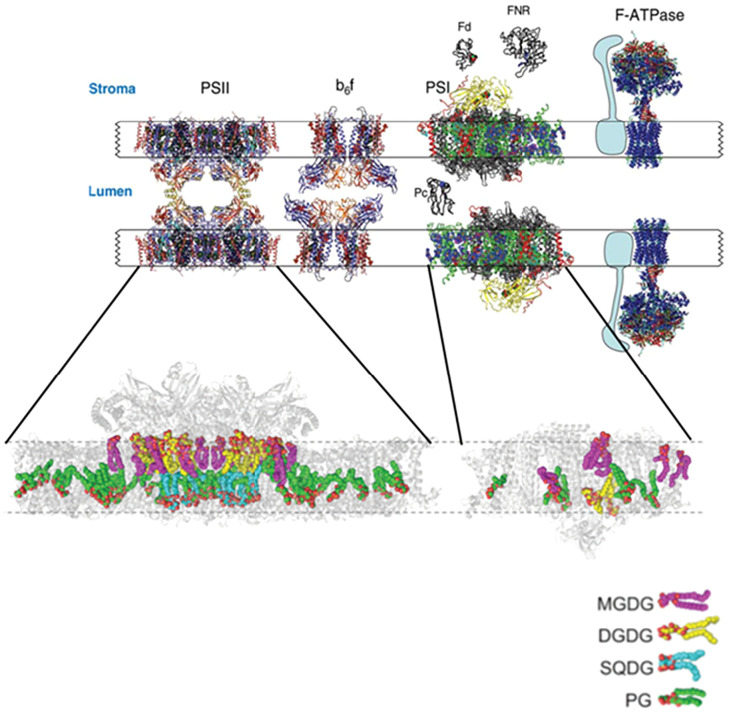
Ultrastructure of thylakoid membranes showing the four major protein complexes (above) and the distribution of the associated glycerolipids (below). Available structural data on membrane–protein complexes and soluble proteins have been adjusted to the relative size of plant photosystem I (PSI). The top part of the figure is taken from Nelson *et al.*, 2006 ^12^ and the bottom section from Yoshihara *et al.*, 2022.^9^

The majority of these lipids (90%) are present in the bilayer regions, but the rest are integral parts of the protein complexes; interestingly, unlike the other thylakoid glycerolipids, MGDG on its own cannot form stable bilayers (lamellar phase) but instead the majority of MGDG molecules form inverted hexagonal (H_II_), or isotropic, or cubic phases within the bilayer continuum^8^ (see Fig. 5). A small proportion of MGDG molecules is held in protein complexes whereas 30 mol% of PG molecules are located in the photosystem complexes (see Fig. 6).^9^

In addition to glycerolipids, thylakoid membranes contain other lipids that tend to exist in their free, non-esterified, forms: these include carotenoid and chlorophyll pigments, and vitamins E (α-tocopherol) and K_1_ (phylloquinone). α-Tocopherol is located mostly in the bilayer membranes, presumably offering structuring and antioxidants roles, with a smaller proportion in the PSII complex, where two molecules of α-tocopherol protect the photosynthetic apparatus from oxidative damage by quenching singlet oxygen (^1^O_2_).^13^

Carotenoids, chlorophyll, and phylloquinone are concentrated in PSI and II, which are each constructed of a central core complex including the reaction centre surrounded by light harvesting complexes (LHC) collectively known as the antennae region, responsible for absorbing sunlight energy and transferring it to chlorophyll at the core of the reaction centre. Phylloquinone is an electron acceptor (A_1_ cofactor) in PSI, where there are 2 molecules of phylloquinone per PS1 complex. Chlorophyll and β-carotene are enriched at the reaction centres of PSI and II, whereas lutein, violaxanthin and neoxanthin are enriched in LHC I and II. Some carotenoids (15 mol%) are also located in the thylakoid bilayer membrane where lutein can increase its stiffness and β-carotene can increase its fluidity.^14^ The relative molar ratio of total chlorophyll (*a* + *b*) to total carotenoids ranges between 3 : 1 to 6 : 1 with 4 : 1 being a common ratio amongst plants. The relative molar ratio of carotenoid type is roughly lutein (45 mol%), β-carotene (25 mol%), violaxanthin (15 mol%), neoxanthin (15 mol%). On a molar basis, chlorophyll is the most abundant pigment molecule in thylakoid membranes, but overall, on a molar basis MGDG is the most abundant lipid (MGDG : chlorophyll molar ratio is between 2 : 1 and 3 : 1).

Plants can be categorised as C3, C4, or CAM (crassulacean acid metabolism); they differ primarily in how they fix carbon dioxide in the chloroplast during photosynthesis. CAM plants (such as cacti, orchids and pineapple) open pores and store CO_2_ at night to photosynthesise during the day; closed pores during the day limit water loss and so these plants are well-suited to arid environments like deserts. C4 plants (such as maize, sugarcane, and sorghum) use a specialised pathway to concentrate CO_2_ in bundle sheath cells and thrive in hot and sunny conditions. C3 plants make up 95% of all plants and algae and are most common in moderate climates. Although these three categories of plant all contain chloroplasts with similar constituents, their actual composition can differ; for example, RuBisCO can make up more than 50% of the chloroplastidial protein in C3 plants, whereas it is only 8–23% of the chloroplastidial protein in C4 plants. Given the abundance of C3 plants, the following account of chloroplast composition is representative of this category of plants, but growing conditions, abiotic stress and age can all affect the composition of chloroplasts.

## 2. A case to consider chloroplasts as ingredients for food

Given the importance of photosynthesis to life on earth, it is not surprising that this tiny, but prolific, organelle has received close scientific attention over the years, but few researchers have viewed these organelles from an applied science/technology perspective; here are some examples. Modifying chloroplast genomes has shown promise in developing crops with enhanced resistance to pests, extreme temperatures, drought, and oxidative stress. In some cases, such modifications may even enable plants to assist in environmental cleanup through phytoremediation. Within food science, chloroplast engineering can contribute to improving the nutritional quality of crops by boosting their mineral, micronutrient, and macronutrient content.^15^ Moreover, the chloroplast's capacity for high-yield protein synthesis has been leveraged to produce valuable biomolecules. Transplastomic plants, for example, have been successfully used to generate therapeutic proteins, industrial enzymes, and vaccines.^16,17^ Another example is the application of thylakoid membranes to suppress appetite,^18^ although further studies failed to show a reduction of body mass in obese subjects. Our group is interested in the composition and properties of chloroplasts in terms of their potential as ingredients in the food, feed and pharmaceutical industries, tapping into their nutritional value and the interfacial properties of the multicomponent thylakoid membranes/chloroplast membrane materials (CMM).

The nutritional composition of a range of green leafy vegetables (GLVs) has been well established.^19–21^ A summary of macronutrients (proteins, lipids and carbohydrates) and micronutrients (vitamins and minerals) in various GLVs (*e.g.*, spinach, kale, cabbage and broccoli) is provided in Table 1.

**Table 1:** Concentrations of macro and micronutrients per 100 g (*F*_wt_) of various raw green leafy vegetables (GLVs)

Species	Protein (g)	Lipids (g)	CHO (g)	Ca (mg)	Mg (mg)	P (mg)	K (mg)	Fe (mg)	Zn (mg)	Cu (mg)	β-Car (mg)	VC (mg)	α-Toc (mg)	VK (mg)
Arugula	2.6^b^	0.66^b^	3.7^b^	160^b^	47^b^	52^b^	369^b^	1.46^b^	0.47^b^	0.08^b^	1.42^b^	15^b^	0.43^b^	0.11^b^
Broccoli	2.8^b^	0.37^b^	6.6^b^	47^b^	21^b^	66^b^	316^b^	0.73^b^	0.41^b^	0.05^b^	0.36^b^	89^b^	0.78^b^	0.10^b^
Chicory	1.7^b^	0.30^b^	4.7^b^	100^b^	30^b^	47^b^	420^b^	0.90^b^	0.42^b^	0.30^b^	3.43^b^	24^b^	2.26^b^	0.28^b^
Coriander	2.1^b^	0.52^b^	3.6^b^	67^b^	26^b^	48^b^	521^b^	1.77^b^	0.50^b^	0.23^b^	3.93^b^	27^b^	2.5^b^	0.31^b^
Lettuce	1.4^b^	0.15^b^	2.8^b^	36^b^	13^b^	29^b^	194^b^	0.86^b^	0.18^b^	0.03^b^	4.44^b^	9^b^	0.22^b^	0.13^b^
Swiss Chard	1.8^b^	0.20^b^	3.7^b^	51^b^	81^b^	46^b^	379^b^	1.8^b^	0.36^b^	0.18^b^	3.65^b^	30^b^	1.89^b^	0.83^b^
Watercress	2.3^b^	0.10^b^	1.2^b^	120^b^	21^b^	60^b^	330^b^	0.20^b^	0.11^b^	0.08^c^	1.91^b^	43^b^	1.00^b^	0.25^b^
Cabbage	0.9^f^	0.02	7.2^f^	20^f^	23^f^	32^g^	53^f^	0.75^f^	0.31^f^	0.05^f^	0.004^g^	30^g^	—	—
		—	—	31^g^	13^g^	—	173^g^	0.30^g^	0.15^g^	—	—	—	—	—
Amaranth	2.5^b^	0.33^b^	4.0^b^	215^b^	55^b^	50^b^	611^b^	2.32^b^	0.90^b^	0.16^b^	1.80^b^	43^b^	—	1.14^b^
	6.00^e^	0.50^e^	6.1^e^	401^e^	223^e^	102^e^	768^g^	3.57^e^	3.06^e^	0.34^e^	6.20^g^	70^c^	—	—
	—	—	—	306^g^	182^g^	64^g^	—	6.00^g^	0.63^g^	—		126^g^	—	—
Spinach	2.9^b^	0.40^b^	3.6^b^	99^b^^,^^g^	79^b^	49^b^^,^^g^	558^b^	2.71^b^	0.53^b^^,^^g^	0.13^b^	5.63^b^	28^b^^,^^g^	2.03^b^	0.48^b^
	2.1^d^	1.20^a^^,^^d^	1.6^d^	141^d^	23^d^	52^d^	776^d^	0.96^d^	0.89^d^	0.10^d^	4.56^d^	—	0.62^d^	—
	—	—	—	—	13^g^	—	—	0.30^g^	—	—	4.03^g^	—	—	—
Kale	4.3^b^	0.93^b^	8.8^b^	150^b^	47^b^	92^b^	491^b^	1.47^b^	0.56^b^	1.50^b^	5.93^b^	120^b^	1.54^b^	0.70^b^
	7.3^d^	3.40^a^^,^^d^	6.4^d^	520^d^	74^d^	151^d^	833^d^	2.44^d^	0.48^d^	0.10^d^	15.40^d^	—	1.80^d^	—

a lipid, which indicates lipid mass relative to dry weight.

b Values taken from Alfino *et al.*, 2016.^21^

c Values taken from Yadav *et al.*, 2013.^22^

d Values taken from Gedi, 2017.^23^

e Values taken from Odhav *et al.*, 2007.^24^

f Values taken from Ashfaq *et al.*, 2018.^25^

g Values taken from Steyn *et al.*, 2001.^26^

Given the nutrient composition of GLVs, their consumption is associated with a healthy diet.^27,28^ The bioavailability of GLV nutrients remains limited due to the food matrix effect, for instance, the encapsulation of biomolecules within cells and food matrices.^29^ By breaching indigestible cell walls, the bioaccessibility, and hence bioavailability, of these nutritional compounds will increase.

### 2.1. Chloroplast-located nutrients

It has long been recognised that plants synthesise fatty acids in plastids/chloroplasts.^30^ Other lipophilic molecules, such as carotenoids, chlorophyll and tocopherols, are also synthesised in plastids.^31–33^ Building on these observations, we recognised that most of the nutrients in GLVs that are of value to human health are located within chloroplasts. Despite these organelles having been a focus of research for many years due to the primary importance of photosynthesis in the biospheres,^34–37^ the fact that the proteins, lipids and micronutrients in a leaf are mostly concentrated in this organelle has been overlooked. From this insight, several benefits emerge from liberating/recovering chloroplasts from GLVs, or underutilised green biomass using a simple physical process:

• The majority of the nutrients contained in the biomass are concentrated into a mass of natural particles

• Nutrients are separated from antinutrients

• The cell wall is no longer present to block digestive enzymes, thus increasing the bioavailability of plant nutrients on ingesting this preparation enriched in chloroplasts

#### 2.1.1. Nutrient roles *in vivo*

**Fig. 7:**
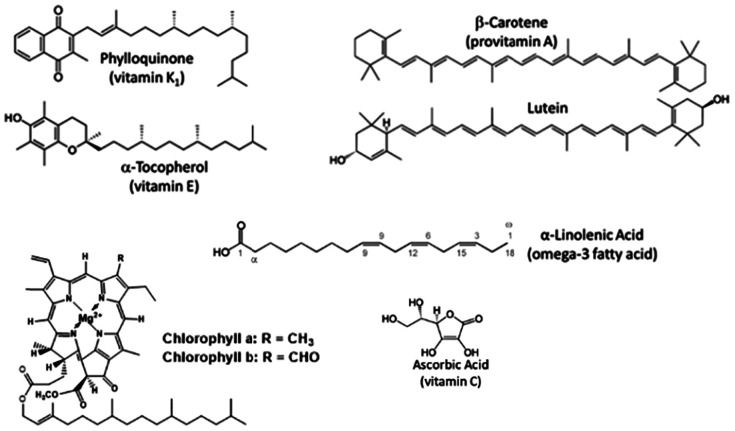
Chemical structure of key organic molecules located in chloroplasts that can be classified as micronutrients.

Below is a list of lipophilic and hydrophilic molecules, found in chloroplasts, of interest as nutrients and their roles *in vivo*. Fig. 7 shows the chemical structure of the organic molecules in this list.

##### Chlorophylls

Chlorophyll a and b are the pigments which the chloroplast, and thus GLVs, owe their vibrant green colour to. In addition to being a source of magnesium (centrally bound in chlorophyll structure),^38^ dietary chlorophyll and chlorophyll-derivatives have been identified as having anti-inflammatory^39^ and antioxidant activity.^40^

##### Carotenoids

There are four carotenoids associated with GLVs : β-carotene (α-carotene is also preset, but at low concentrations), lutein and zeaxanthin/violaxanthin (part of the xanthophyll cycle), and neoxanthin.^41^ α-Carotene and β-carotene are precursors to vitamin A, which is essential for rhodopsin production (a light sensitive protein in the eye rod cells) and proper gene regulation mediating growth and development.^42^ Deficiency is most strongly linked with xerophthalmia (dry eye syndrome) and night blindness.^43^ Lutein and zeaxanthin also play key roles in eye health, protecting from low and high light strain respectively through absorption of blue light^44^ – with insufficient levels in the diet being linked to age related macular degeneration.^45^ Additionally, GLV carotenoids have been shown to support immune response,^46^ brain function –in young adults^47^ and Alzheimer's patients^48^ – and inflammation markers^49^ through their antioxidant and membrane stabilising properties.

##### α-Tocopherol (vitamin E)

α-Tocopherol is the major isoform of vitamin E in the human body and is transported from the liver *via* the bloodstream to other tissues, where it retains its fat-soluble antioxidant function.^50^ Known to be essential for reproductive health,^51,52^ other positive effects of α-tocopherol consumption have been shown in bone and cardiovascular diseases, as well as diabetes.^53^

##### Phylloquinone (vitamin K1)

Phylloquinone belongs to the Vitamin K family, known specifically as Vitamin K_1_ – with the *K* originating from the German word for coagulation (Koagulation) due to this vitamin's essential role in the production of blood clotting factors.^54^ It also plays a role in connective tissue calcification, linking it to potential treatments of osteoporosis and heart disease.^55^

##### α-Linolenic acid

α-Linolenic acid is an omega-3 fatty acid and functions as a precursor to several plant aliphatic compounds, such as membrane glycerolipids (which maintain and regulate membrane function) and jasmonates (hormone regulators of plant development and stress response, which are synthesised in the chloroplasts).^56,57^ For humans, α-linolenic acid is an essential fatty acid that must be gained from dietary sources. It is the precursor of very-long-chain ω-3 polyunsaturated fatty acids,^167^ the consumption of which is associated with various health benefits, such as a reduced risk of cardiovascular mortality,^56^ improved infant visual development,^57^ and a reduction in inflammation for rheumatoid arthritis patients.^58^

##### Ascorbic acid (vitamin C)

Ascorbic acid, more commonly known as vitamin C, functions in the chloroplast to detoxify reactive oxygen species (ROS) produced during periods of plant stress such as drought, heat, or high salinity.^59^ Under excess light, it acts as an electron donor to prevent ROS formation.^60^ Due to its detoxifying and co-factor ability, ascorbic acid also contributes to the plant defence response against fungal and bacterial infection^61^ – much like its role in the human immune system. Vitamin C is shown to support the function of both the innate – due to antioxidant properties and promotion of neutrophil activity,^62^ and the adaptive immune system – by enhancing lymphocyte proliferation and antibody levels.^63^ Vitamin C acts as a cofactor for enzymes involved in collagen synthesis; deficiency results in scurvy, which is strongly associated with increased risk of infection.^64^

##### Iron

Iron is essential within all photosynthetic apparatus that mediates electron transport – photosystems II (PSII) and I, the cytochrome b_6_f complex, and ferredoxin-due to its role within redox reactions and as a protein co-factor.^65^ Iron is most concentrated in the chloroplast out of all leaf organelles,^66^ with its deficiency in plants linked to reduced chlorophyll content and photosynthetic performance.^67^ Whereas in humans, iron deficiency is most linked to anaemia – a reduction in the body's ability to produce red blood cells and thus circulate oxygen. Around 2 g of the body's iron is in the form of red blood cell haemoglobin.^68^

##### Manganese

Manganese ions exist as a cluster within the oxygen-evolving complex associated with PSII. Through light driven loss of electrons the manganese cluster is capable of splitting a highly stable water molecule into oxygen – released as a byproduct – and protons – which are essential for ATP synthesis.^69^ Crucial for photosynthesis and oxygen production in plants, manganese is also necessary for a wide range of normal metabolic and enzymatic functions in humans, such as development, immune and nervous system, reproductive hormone activity, and energy metabolism.^70^ Humans are rarely manganese deficient; however, despite being rarely encountered, excessive consumption can lead to neurotoxicity known as manganism leading to Parkinson's disease like symptoms.^71^ Although recent studies have called into question the safe maximum of 11 mg day^−1^ (particularly in the case of infants drinking re-constituted milk formula), it is unlikely that average diets would come close to this value even when consuming CRF.^72^

## 3. The composition and nature of chloroplast-containing powders

### 3.1. Developing methods to prepare dry powders that contain chloroplasts released from plant cells

Many methods have been used to isolate intact chloroplasts and thylakoid membranes; the choice of the method depends on the nature of the study. Most published methods have been developed to study the process of photosynthesis, necessitating the preservation of the complex machinery that is activated during photosynthesis. One assumption is that chloroplasts are osmotically sensitive and will burst when released into water. Most traditional methods of chloroplast isolation have therefore used an osmoticum, such as cold isolating buffer^73,74^ and sucrose solution^75,76^ in the grinding medium to prevent chloroplasts from bursting. After this initial extraction step (*i.e.*, cell disruption), it may also be necessary to extract and/or purify the chloroplasts using, for example, sequential centrifugation,^77^ density gradient and/or continuous Percoll gradient centrifugation.^74,78^ All these steps require a cool temperature and elimination of light, if the photosynthetic machinery is to remain intact. Another target for chloroplast extraction/isolation, in addition to preserving organelle structure, is protecting components from the action of endogenous catabolic enzymes, for example, lipolytic and proteolytic enzymes; their activity is limited by low temperature and careful handling of the plant material.

**Fig. 8:**
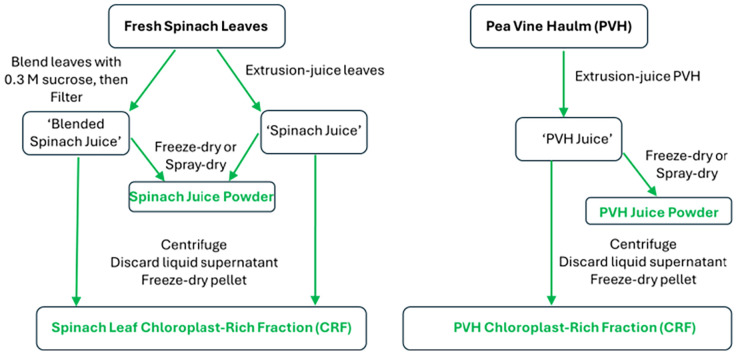
Processes to release and stabilise chloroplasts from the cells of green biomass. From here ‘CRF’ refers to a freeze-dried pellet enriched in chloroplasts, unless qualified as ‘fresh CRF’ which refers to the pellet enriched with chloroplasts prior to freeze-drying.

**Fig. 9:**
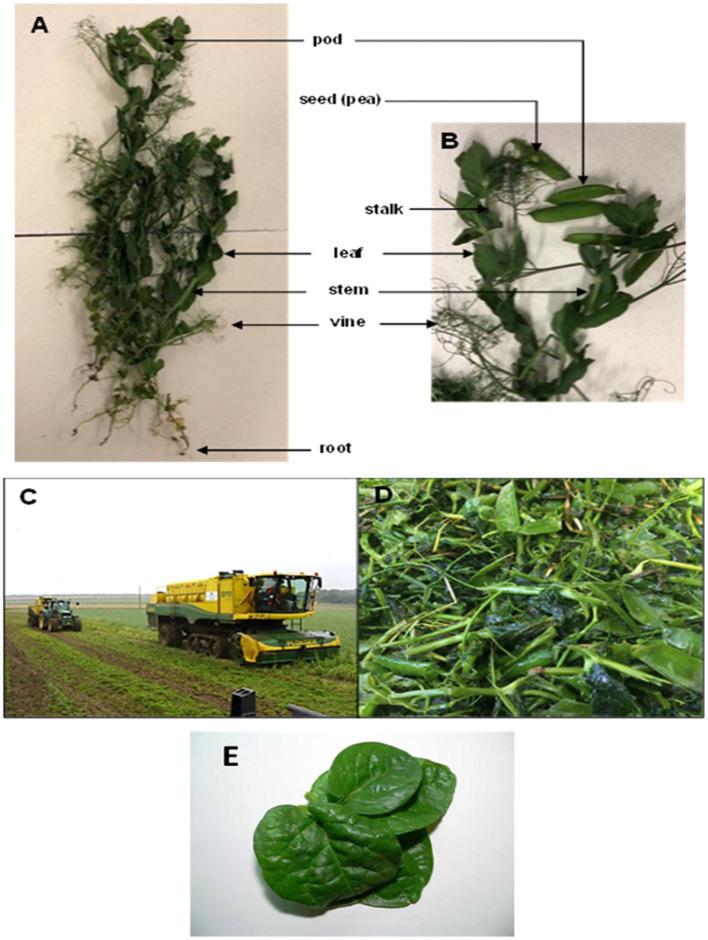
Post harvest pea vine haulm and spinach leaves. (A and B) morphology of pea plant (C) during harvesting (D) postharvest, pea vine field residue (haulm) (E) spinach leaves. Images A–D are taken from Wattanakul, 2020.^80^

Designing a commercial process to make chloroplast preparations as ingredients for food or other industries does not require the exacting extraction and purification protocols employed by plant biochemists; also, crude preparations of chloroplasts are preferred over pure preparations to avoid costly separation steps. The commercial process would have to limit the loss of nutrients, and/or retain the structure, if not the photosynthetic ability, of the multicomponent thylakoid membranes. We have tested lab-scale methods to extract chloroplasts that could be scaled up easily for industry. Initially, we used published methods, using sucrose as an osmoticum during a leaf-blending stage.^76^ Then we established the nutritional credentials of chloroplasts: chloroplast-rich fractions from spinach, kale, nettles and grass all contained high levels of proteins and lipids, and also notable concentrations of the micronutrients α-tocopherol (vitamin E), β-carotene (pro-vitamin A), lutein, Fe and Mn. In a step to reduce water and sugar usage, we hypothesised that chloroplasts, if kept in their natural environment, would remain intact. A simple juicing step expels cellular organelles into their cytoplasmic milieux, with some dilution caused by the rupture of any attendant vacuoles. From our work, juicing appears to retain chloroplast integrity, at least as a particulate that can be further concentrated. Fig. 8 summarises the methods we have developed to release chloroplasts from the cells of green biomass: spinach leaves (well characterised and available throughout the year) and pea vine haulm (PVH, the postharvest field residue from pea vining, a potentially cheap and sustainable source of chloroplasts) are the green plant materials we have focused on (Fig. 9).

Table 2 summarises the nutritional composition of a range of chloroplast-rich powders derived from spinach leaves or PVH. All CRF materials were prepared, as shown in Fig. 8, by freeze-drying a pellet generated by centrifuging juice. Juice, on the other hand, was either freeze-dried (FD) or spray-dried (SD) to produce a ‘juice’ powder. Spray-drying of juice was carried out without any carrier such as maltodextrin, and the particles were not altered by any further physical processes. The freeze-dried material (juice or CRF) was ground using a mortar and pestle and then sieved to collect the particles that were less than 250 µm in size (*i.e.* 50 times bigger than the average size of chloroplasts from higher plants (5 µm)).^79^ The benefit of centrifuging the juice is that the nutrients are enriched in CRF compared with the dried juice (Table 2), and any water soluble/dispersible antinutrients (such as phytates and tannins, if present in the plant biomass) should remain in the supernatant phase and so not be present in the CRF. It is worth noting at this stage that both the spray-drying and freeze-drying processes retained chloroplast nutrients well (Table 2) but resulted in differences in the structure and physical properties of the powder particles, which are further explored in section 3.3. [See SI for a comparison between some of this data and the nutritional composition of algae, often sold as health supplements. The nutritional composition of CRF compares favourably with unicellular algae such as *chlorella* and *spirulina*.]

**Table 2:** Nutrient composition of spray- or freeze-dried juice or freeze-dried pellet (CRF) derived from spinach leaves and pea vine haulm

Nutrients measured across a range of studies over several years unit = mg per g dry wt mean (range of values)	Spinach leaf				Pea vine haulm	
	Spinach juice from waring blended leaves		Spinach juice from extrusion juicing		PVH juice	
	(Blend leaves in 0.3 M sucrose) spinach leaf CRF	(Blend leaves in ultrapure water) water-burst CRF (WBCRF)	Dried spinach juice powder (SD or FD)	Spinach leaf CRF	Dried PVH juice powder (SD or FD)	PVH CRF
Protein	**458** (400–550)	**600**	**449** (355–537)	**509** (446–596)	**298**	**321** (205–497)
Lipid	**337** (305–396)	**322**	**114** (97–130)	**264** (239–269)	**80**	**150** (90–223)
Carbohydrate	**164**	**32**	**306** (198–414)	**118** (96–139)	**ND**	**526**
Ash	**41**	**43**	**168** (100–208)	**62** (51–72)	**ND**	**30**

Chlorophyll	**53.2** (21.2–70.0)	**70**	**12** (8.5–17.9)	**49.2** (35.5–66.0)	**1.2**	**14.6** (9.3–18.5)
% chlorophyll relative to total lipid	**15.8%**	**22.0%**	**14.0%**	**18.6%**	**1.5%**	**9.7%**
β-Carotene	**4.3** (1.2–7.0)	**6**	**0.6** (0.2–1.2)	**3.4** (1.4–5.3)	**0.7**	**1.0** (0.16–2.8)
Lutein	**4.1** (3.4–4.8)	**12**	**0.9** (0.7–1.2)	**3.7** (2.1–4.7)	**1.1**	**2.3** (0.4–4.8)
β-Carotene + lutein	**4.4**	**18**	**1.5**	**4.1**	**1.8**	**3.3**
% β-Carotene + lutein relative to total lipid	**1.3%**	**5.6%**	**1.7%**	**2.7%**	**2.3%**	**2.2%**
α-Tocopherol	**0.4** (0.2–0.6)	**0.6**	**0.2** (0.15–0.23)	**1.2** (0.5–4.0)	**0.1**	**0.4** (0.3–0.5)
Phylloquinone	**2.8**	**ND**	**ND**	**2.6**	ND	**0.67** (0.64–0.70)
α-Linolenic acid	**36.3** (25–50)	**ND**	**10.9** (9.7–12.1)	**36.9** (31–43)	**2.2**	**11.2** (4.5–16.7)

Ascorbic acid	**3.6** (0.8–6.4)	ND	ND	**2.2** (1.5–2.8)	**0.3**	ND
Mg	3.4	ND	ND	2.8	ND	1.5
Fe	0.3	ND	ND	0.9	ND	2.1
Mn	0.08	ND	ND	0.1	ND	0.07

### 3.2. Nutritional value of chloroplast powders prepared from pea vine haulm and spinach leaves

Both dried juice and dried pellets (CRF) contain a wide range of nutrients, including some vitamins, that are concentrated compared with the fresh weight by the removal of water (often representing 80–90% of the fresh weight) (Table 2). Concentrating the chloroplasts dispersed in the juice by centrifuging to form a pellet, further enriches/concentrates the nutrients. Table 3 shows the % adult Nutrient Reference Intake (NRI) for selected micronutrients that 5 g (approximately a teaspoon) of dried juice or dried pellet (CRF) derived from spinach leaves or PVH can contribute to the adult human diet.

**Table 3:** Nutritional value of dried juice and CRF from green materials

Micronutrient	Nutrient Reference Intake (NRI) for adults (mg day^−1^)	Spinach leaf				Pea vine haulm (average 2016/2017 harvests)			
		Juice		CRF		Juice		CRF	
		mg per 5 g dried sample	%NRI per 5 g dried sample	mg per 5 g dried sample	%NRI per 5 g dried sample	mg per 5 g dried sample	%NRI per 5 g dried sample	mg per 5 g dried sample	%NRI per 5 g dried sample
β-Carotene (provitamin A)	3.6	12.7	**>100**	23.5	**>100**	3.3	**91.7**	9.6	**>100**
Lutein	10	11.4	**>100**	22	**>100**	5.7	**56.5**	24	**>100**
α-Tocopherol (vitamin E)	15	1.1	7.3	3	**20**	0.6	4	2	**13**
Ascorbic acid (vitamin C)	40	ND	ND	7.7	**19.3**	1.4	3.4	1	2.5
Phylloquinone (vitamin K1)	0.06	ND	ND	13	**>100**	ND	ND	3.3	**>100**
α-Linolenic Acid	1600	ND	ND	156	**10.0**	10.8	0.7	80	5
Iron	12	ND	ND	4.4	**36**	ND	ND	11	**88.3**
Manganese	2.3	ND	ND	0.6	**26**	ND	ND	0.4	**15.2**
Magnesium	300	ND	ND	14	4.6	ND	ND	7.7	2.6

Nutrients in CRF are more concentrated than in dried juice, as expected, and CRF from PVH has a similar nutrient profile and concentration as CRF from spinach leaves. The higher iron content in PVH CRF compared with spinach CRF may be explained by the carry-over of soil-derived iron onto the PVH during sample collection; however, one may anticipate a commensurate increase in magnesium in the PVH CRF material if soil contamination is the reason, but this is not the case. Protein values for spinach and PVH juice and CRF powders range from 30–60% (Table 2). If the protein requirement for an adult is 40–60 g per day, then 5 g of these powders will, at best, contribute 8% to the daily protein requirement of an adult. The presence of nucleic acids in chloroplasts suggests a dietary hazard for those suffering from hyperuricemia, a common form being ‘gout’, so it may be expedient to limit intake of these powders to 5 g per day, an amount that clearly provides a significant source of pro-vitamin A, lutein, vitamin E, vitamin K_1_, α-linolenic acid (an omega-3 fatty acid), iron and manganese (Table 3). Ingesting intact chloroplasts in quantities that supply the dietary requirement of a range of micronutrients may not supply significant amounts of protein, but if the quality of the protein in these powders is high, there would be a case to fractionate the chloroplast into the chloroplast membrane material (CMM), and the protein-rich stromal fraction.

**Fig. 10:**
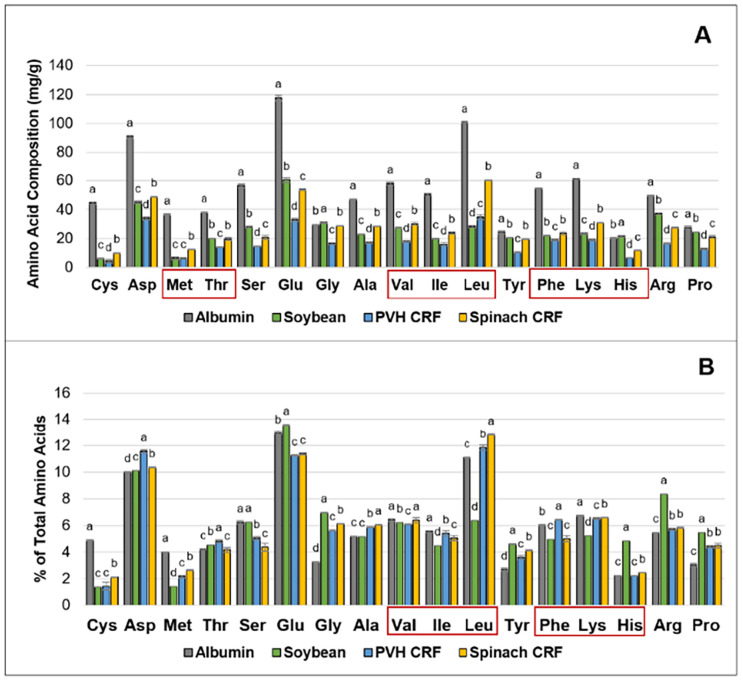
Amino acid composition of albumin, soybean, PVH CRF and spinach CRF. (A) mg g^−1^, DW (B) proportion of each amino acid expressed as a percentage of total amino acids. Values for soybean were taken from ref. 83. Data were presented as a mean ± SD of 3 separated samples and analysed using *post-hoc* analysis of variance (ANOVA) and according to a Tukey test with statistical significance at *p* < 0.05, *a* > *b*; *a* was a higher value than *b* followed by *c* and *d*, respectively; Cys cysteine, Asp aspartic acid, Met methionine, Thr threonine, Ser serine, Glu glutamic acid, Gly glycine, Ala alanine, Val valine, Ile isoleucine, Tyr tyrosine, Phe phenylalanine, Lys lysine, Leu leucine, His histidine, Arg arginine, Pro proline. Essential amino acids are highlighted in red boxes. Figure taken from Wattanakul, 2020.^80^

The amino acid (AA) composition of spinach and pea vine CRF was compared with high quality protein sources from both animal (albumin) and plant (soybean) origin (Fig. 10). The AA content of the CRF from spinach leaves and PVH was substantially lower than albumin but exhibited an amino acid profile comparable to soybean, a well-established plant protein benchmark. Spinach CRF contained slightly higher essential AAs than pea vine CRF and in some cases even surpassed soybean. It was particularly rich in branched chain amino acids (leucine, isoleucine, valine), which play key metabolic roles in the body, and in lysine, an essential AA lacking in cereals. Given these nutritional advantages, spinach CRF holds promise for developing functional food products with targeted metabolic benefits.

The stromal protein RuBisCO is the most abundant protein in chloroplasts; in C3 plants it makes up 50% by mass of all proteins in this organelle. It is rich in essential amino acids, and its large-scale extraction directly from green biomass was noted in 2011 ^84^ and is now a commercial reality. What has not been developed yet is a commercial process where chloroplasts are released from green biomass and used directly in applications or are further fractionated into CMM and a stromal fraction; this would increase the number of products gleaned from green biomass, adding to the economic case for recovering chloroplasts from green biomass. In addition to nutritional value, proteins in food can provide functional benefits such as emulsifying power, foam stabilisation and gelation. If RuBisCO (or a crude stromal protein preparation) were fractionated from CRF, then it could be marketed for its nutritional value and/or its functional properties.

A strong candidate for protein-rich stromal fractioning would be members of the Lemnoideae family, more commonly referred to as duckweeds. Interest in duckweeds as a novel food crop has increased due to its fast growing rates, regularly doubling biomass in less than two days,^85^ and high protein content, ranging from 20 to 35% of freeze-dried weight.^86,87^ Wholly consisting of leaf-like structures, duckweed lends itself well to the methods we have presented – a duckweed extract has been generated with 67% protein content.^88^ Recent acceptance of duckweed-based protein concentrates by the European Commission^89^ furthers the attractiveness for a commercially optimised method to extract duckweed protein.

### 3.3. Physical nature of powders from fresh spinach juice or a spinach chloroplast-rich fraction (CRF)

In addition to measuring the composition of the chloroplast-rich materials/powders that we generated, we investigated their physical nature. Particle size and morphology, and their solubility in water, were measured/observed. These physical properties provide information about the powders that is relevant when incorporating them into food/pharma formulations. We also measured the moisture content and water activity of our chloroplast-rich materials/powders; these parameters provide an indicator of the likely shelf-life of these materials. In general, as Aw (water activity) increases so does microbial growth and endogenous enzyme activity. Some chemical reactions that affect the quality of food materials have a more nuanced association with Aw. For example, lipid oxidation tends to be low at intermediate water activities and higher at low and high Aw values.^90^ Chloroplasts are rich in omega-3 fatty acids, making them vulnerable to oxidation; however, chloroplasts are also rich in carotenoids and tocopherol, which can inhibit photo- and auto-oxidation, respectively. It is important to note that the freeze-dried material (juice or CRF) formed one solid mass after the drying step, so it was ground using a mortar and pestle and then sieved to collect the particles that were less than 250 µm in size. Spray-drying juice (SJ) resulted in a powder that does not require any sieving.

The volume mean diameter (*D*_4,3_) of spinach juice powder was significantly affected by drying techniques: 9.81 μm for SJ and 66.44 μm for freeze-dried juice (FJ) (Table 4). These results are in agreement with those reported for Brazilian ginseng root powders obtained by spray- and freeze-drying, with *D*_4,3_ values of 9 and 207 μm, respectively,^91^ which suggests that spray-dried powders normally contain particles with a smaller size than freeze-dried powders. The *D*_4,3_ of spinach CRF powders (FC) was nearly twice that of FJ; both FC and FJ were ground using a mortar and pestle then sieved through a 250 µm mesh, so the smaller size of freeze-dried juice (FJ) particles compared with FC (CRF) particles suggest that the solid created after freeze-drying the juice is more fragile/brittle than the solid created after freeze-drying the concentrated pellet.

**Table 4:** Particle size of powders from spinach juice or a chloroplast-rich pellet (μm)

Parameter	SJ	FJ	FC	Fresh chloroplast pellet
*D*_4,3_	9.81 ± 0.22^c^	66.44 ± 0.38^b^	113.95 ± 4.44^a^	10.01 ± 0.60^c^
*d*_10_	2.26 ± 0.18^c^	9.26 ± 0.47^b^	19.23 ± 1.29^a^	2.39 ± 0.02^c^
*d*_50_	7.63 ± 0.06^c^	48.75 ± 0.48^b^	96.46 ± 2.62^a^	6.90 ± 0.23^c^
*d*_90_	20.23 ± 1.01^c^	151.62 ± 1.81^b^	215.93 ± 2.88^a^	19.10 ± 1.54^c^
Span	2.36 ± 0.12^b^	2.92 ± 0.07^a^	2.04 ± 0.10^c^	2.42 ± 0.14^b^

**Fig. 11:**
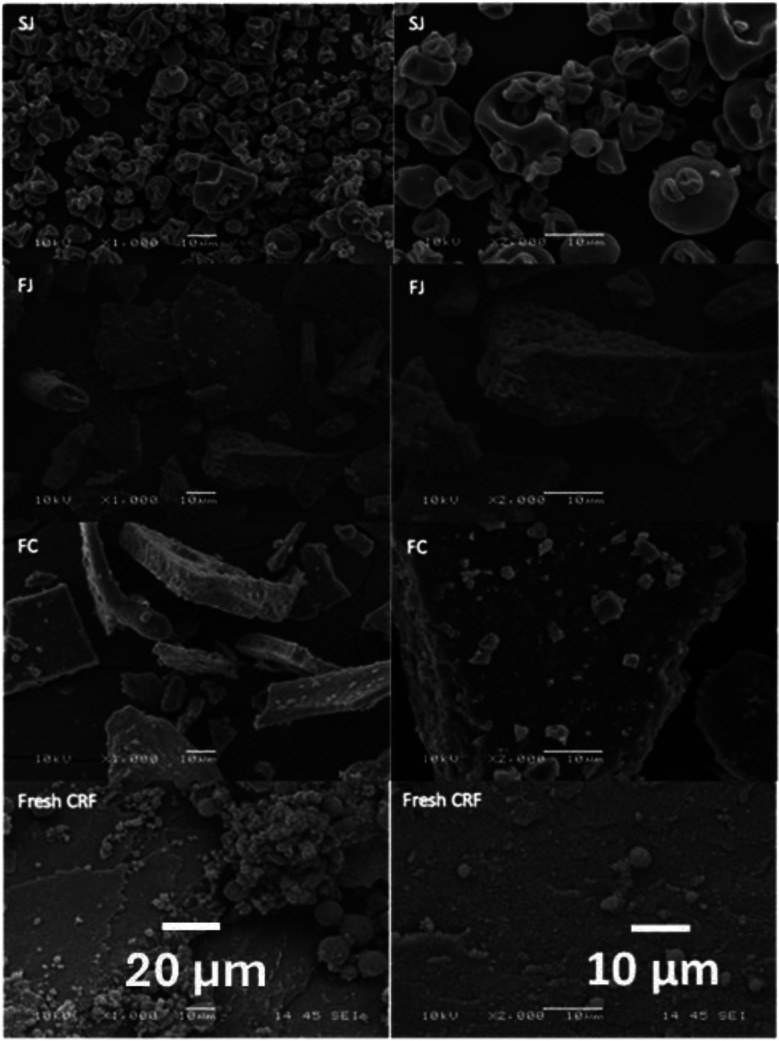
Scanning electron microscope micrographs of spinach chloroplasts. Image taken from Chi, 2023.^82^

The morphology of the particles that make up SJ, FJ, FC and fresh pellet/CRF was studied using light microscopy and scanning electron microscopy (SEM). Light micrographs of these materials suspended in distilled water revealed individual green organelles or small clusters in all cases. The diameter of individual chloroplasts ranges between 4 and 10 μm, which agrees with values quoted in the literature.^92^ These findings confirm that intact chloroplasts can be released from cells by juicing and preserved after both spray-drying and freeze-drying. SEM reveals the shape of these materials in their native state (not resuspended in water) and at a higher resolution (Fig. 11). The SEM images of SJ powders (no added carrier) indicate irregularly spherical shaped particles with many shrinkages and dents on their surface (Fig. 11). Given the abundance and size of these dented objects, it seems likely that they are chloroplasts. Surface dents result from a complex interaction between various factors, such as capillary forces, inlet temperature, drying rate, rapid wall solidification, and uneven shrinkage at early stages of drying.

Chloroplast powders obtained by freeze-drying (FJ and FC) had irregular shaped particles with a tabular habit, which are typical characteristics of freeze-dried products. This phenomenon results from ice crystals forming inside the material during processing, and this helps prevent the shrinkage and collapse of the organelle structure.^93^ Similar structures have been reported for freeze-dried mango and açaí pulp.^94,95^ The particle size of FJ and FC, as shown in the micrographs, was higher than SJ and fresh pellet/CRF, which is consistent with the particle size distribution in Table 4.

In addition to particle size and morphology, some other physical properties of dried spinach chloroplast materials were measured (Table 5). Moisture content in food products and ingredients can significantly affect their quality, shelf-life and spoilage by microorganisms. Fresh pellet/CRF had a high moisture content (83.9 ± 0.2 g per 100 g wet mass), which could promote food degradation and growth of microorganisms and, therefore, reduce shelf life. The moisture content of spray-dried and freeze-dried juice was around 9% (wet mass); moreover, the moisture content of freeze-dried CRF was 4.0% (wet mass). The recommended moisture content for fruit and vegetable juice powder products needed to reduce microbiological growth and to enhance stability of products for long-term storage is 5% (wet mass).^96^

**Table 5:** Some physical properties of powders from spinach juice or a spinach chloroplast-rich fraction (CRF)

Property	SJ	FJ	FC	Fresh chloroplast pellet
Moisture content/% by mass	8.8 ± 0.7^b^	8.5 ± 0.3^b^	4.0 ± 0.8^c^	83.9 ± 0.2^a^
Water activity	0.26 ± 0.01^b^	0.18 ± 0.02^c^	0.24 ± 0.02^b^	1.00 ± 0.00^a^
Solubility/% by mass	63.2 ± 0.4^b^	73.9 ± 1.2^a^	42.6 ± 2.4^c^	41.0 ± 4.6^c^

Water activity (Aw) is measured on a scale of 0–1, where 1 represents pure water, where all water molecules are free and not bound to other molecules. Free water is necessary for most biochemical reactions to occur.^97^ Water activity above 0.3 is a predictor of a shorter shelf life, since more free water is available for deteriorative reactions such as enzymic browning and lipid oxidation.^96^ The water activity found in fresh CRF was 1, equivalent to that of distilled water; this high value of water activity promotes biochemical reactions and supports the growth of bacteria, yeasts, and moulds. The water activity of CRF after freeze-drying significantly decreased to 0.24 ± 0.02. SJ, spray-dried juice, had a slightly higher water activity than FJ, freeze-dried juice (0.26 and 0.18, respectively); this result is consistent with the water activity values of 0.23 for spray-dried and 0.18 for freeze-dried spinach juice obtained by Syamila and co-workers.^98^ Interestingly, FC (freeze-dried CRF) had a higher water activity but a lower moisture content than FJ (freeze-dried juice); this could be due to the higher proportion of soluble carbohydrates in FJ, binding water molecules and reducing the water activity.^99^

Solubility is a fundamental physical property/characterisation of powder products, and powders used as food ingredients must, in general, exhibit good solubility.^91,100^ Solubility is defined as the ability of powders to form a solution or suspension in water.^96^ Powder solubility, in this case, was measured according to the method proposed by Anderson *et al.*^101^ to assess the solubility of food material during digestion, without the influence of enzymes. Powder samples (0.5 g) were added to 14 mL of ultrapure water and vortexed for 1 min, then heated to 37 °C and rotated at 250 rpm for 30 min. The suspension was then transferred to a tube and centrifuged at 3500 rpm at 4 °C for 20 min. The supernatant was transferred completely to a pre-weighed aluminium can and dried at 105 °C for 4 h. The dried soluble solid was then weighed and used to calculate the solubility as a percentage of the total mass.

Solubility values of juice dried by spray-drying or freeze-drying [SJ and FJ, respectively] ranged between 63.2 and 73.9%, significantly higher than that of chloroplast pellet material [freeze-dried (FC in this case) or fresh], which had a tight range of between 41.0 and 42.6% (Table 5). It seems that the composition of the material, more than the drying method determines the % solubility in this case. Juice contains significantly more carbohydrate and ash compared with CRF (Table 2); this appears to render the powder more ‘soluble’ than the more concentrated chloroplast preparations (fresh and freeze-dried).

As we have seen in this section, the method of chloroplast powder preparation clearly affects the physical properties of the particles. We have also observed that both spray-drying and freeze-drying preserve chloroplast-located nutrients to a high degree, and that these nutrients remain relatively stable for weeks when these powders were stored in light-tight pouches at 4 °C or 20 °C.^98,102^ To maximise the preservation of nutrients between harvest and the preparation of chloroplast-rich powders, it would be prudent to include a heat-treatment step postharvest to limit biochemical changes (*e.g.* lipolytic or proteolytic) associated with deterioration reactions. In the next two sections, we explore the impact of heat-treating the biomass or juice on the composition and physical nature of the chloroplast-rich powders.

## 4. Impact of blanching biomass on the yield and composition of the CRF

Our early work on chloroplasts as food/feed ingredients focused on effective ways to release chloroplasts that retain their structure and biochemical composition. This process (Fig. 8) resulted in stable, dry powders with a nutritional composition very similar to that of the fresh chloroplast material. These powders retained their nutrients over extended storage under suitable conditions. At this point, there was no drive to include a heat process whilst generating our powders. However, if the conversion of underutilised green biomass to chloroplast powders were to become a commercial reality, then the supply chain may necessitate a heat-stabilising step, either of the starting biomass or of the juice derived from fresh biomass. The nutrient composition of leaves changes after harvesting due to enzymatic activities^103^ and abiotic stresses. For instance, up to 56% of carotenoids in spinach whole leaf material are lost after storage at 20 °C for 4 days.^104^ Multiple studies have observed a plant's response to different abiotic stresses and how these responses affect the nutritional composition of the green biomass, *e.g.* light,^105^ cutting^106^ and heat stress.^107,108^

**Fig. 12:**
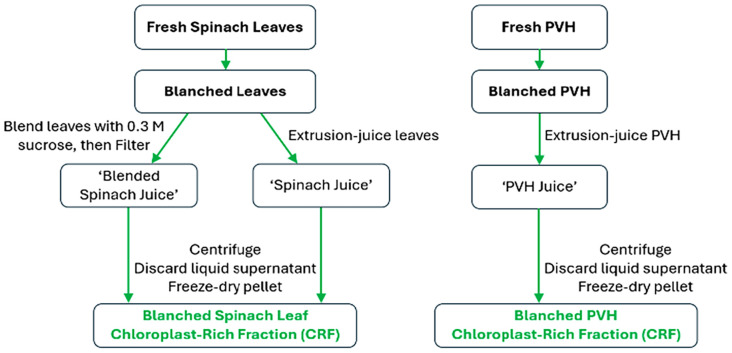
Outline of blanching spinach leaves or PVH prior to preparing CRF.

We tested the impact of heating the biomass prior to juicing on the yield and quality of the derived chloroplast-rich fraction (CRF). Fig. 12 outlines the process we used to heat-treat biomass and to prepare CRF powders from heat-treated biomass.

### 4.1. Impact of blanching spinach leaves on intracellular morphology, peroxidase (POD) and lipolytic activities

**Fig. 13:**
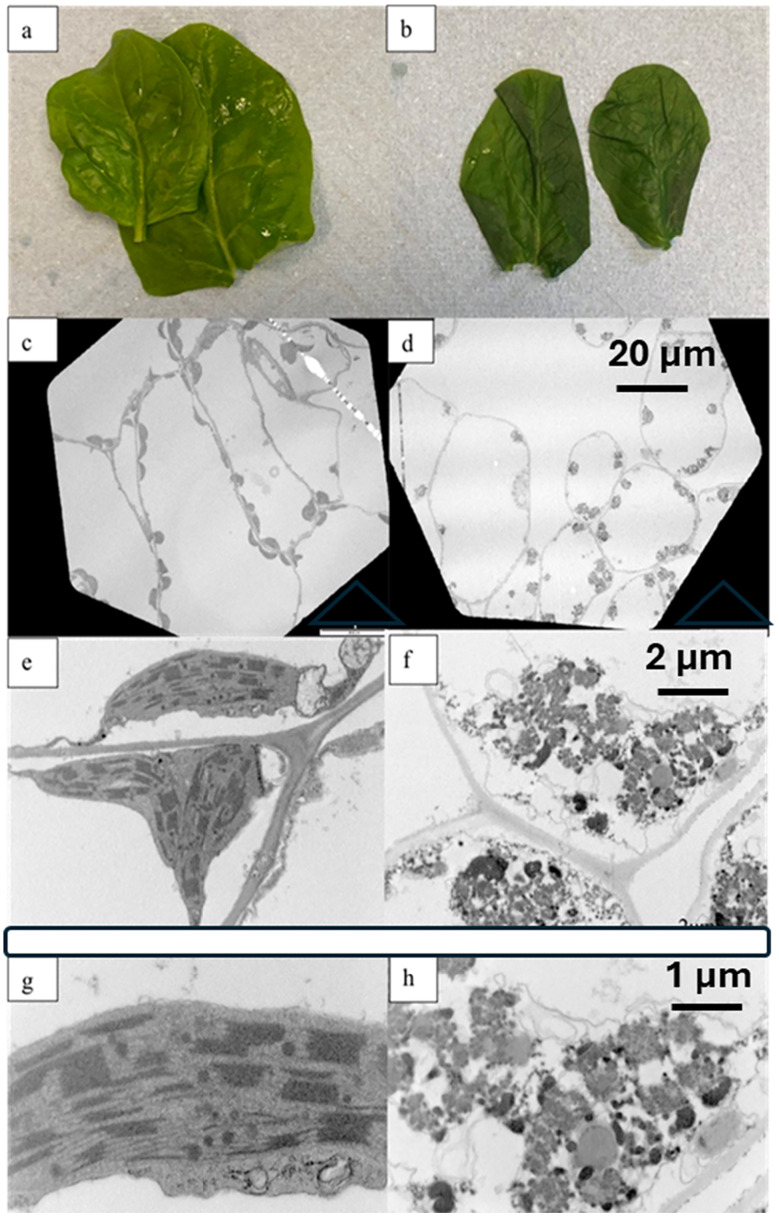
Images of fresh/blanched spinach leaves at different magnifications. (a) Fresh spinach leaves; (b) blanched spinach leaves (100 °C 30 s); (c), (e) and (g) fresh leaves at TEM magnification of 800×, 8200× 27 000× respectively; (d), (f) and (h) blanched leaves at TEM magnification of 800×, 8200× 27 000× respectively. Images taken from Chi, 2023.^82^

Morphological examination of blanched spinach leaves (100 °C water for 30 seconds, then chilled) revealed that heat treatment caused a significant change in the internal architecture of cells (Fig. 13). In fresh leaves, the intact structures of chloroplasts could be seen: cell walls, thylakoids, assembled stacks of grana, and plastoglobuli. Chloroplasts in fresh leaves maintained their integrity with some lens-shaped chloroplasts (3–10 µm in diameter and 1–3 µm thick) around the edges of cell, or slightly twisted bands at the cell edges.^109^ Following blanching, the integrity of the chloroplast's fine ultra-structures was largely destroyed, leaving cell contents indistinguishable and disorganised. This morphological change in itself may not affect the composition and properties of a powder derived from blanched spinach leaves, but the yield of juice from blanched spinach leaves was very low due to the slippery texture of the blanched spinach leaves. This was overcome by blending the leaves in the presence of 0.3 M sucrose (Fig. 12). The juice prepared from blanched spinach leaves in this way had negligible POD activity (a measure of general enzyme activity in plant material), and the CRF prepared from blanched spinach leaves blended in 0.3 M sucrose had intact galactolipids with very low levels of free fatty acids compared to CRF prepared from fresh leaves, indicating the heat-inactivation of endogenous lipolytic enzyme activities.^80,82^

### 4.2. Impact of blanching PVH on the yield and composition of the CRF

Initial work on liberating chloroplasts from PVH tested the impact of wilting (for one week in the dark at RH (relative humidity) values of 33%, 65% and 93%) and fermenting (for one week in the dark in a sealed bag at room temperature) as well as blanching (steamed for 7 min, and cooled for 5 min) on the composition of CRF derived from PVH.^110^ Wilting and fermenting both resulted in significant losses of lipophilic nutrients. This finding underlined the need to process PVH soon after harvest if the objective was to obtain cell-wall free material rich in the nutrients located in chloroplasts. PVH was blanched by bagging 500 g quantities of PVH fresh from the field, heating it up to 100 °C in a steam steriliser, and then holding it for 5 min. The mass was then plunged into ice cold water. Interestingly, unlike blanched spinach leaves, blanched PVH did not ‘slip’ in the extrusion juicer, and therefore, the yield of juice was higher than that from spinach leaves. PVH is more fibre-rich than cultivated spinach leaves; the fibre provides an effective material for the extrusion screws to work against, resulting in biomass cell breakage and the release of chloroplasts and other intracellular entities.

We have observed that blanching biomass before juicing does not cause any significant loss in the micronutrient concentration of the derived CRF preparation; blanching of biomass also allows the retention of CRF-micronutrients that are lost after freezing biomass before juicing. But we have also shown a reduction in the yield of juice when biomass is blanched prior to juicing. Another consideration when optimising the process to prepare chloroplast-rich powders is whether there is a need to retain the native form of thylakoids with their attendant surface-active properties (section 7); it is clear from our work that heat stress of leaves alters the physical nature of thylakoid membranes (Fig. 13). Given the impact on juicing yield and thylakoid integrity, we would not recommend the heat-treatment of biomass prior to the recovery of chloroplasts. If it is necessary to arrest endogenous enzymes for the quality and yield of the chloroplast product, then it would probably be better to pasteurise the juice from fresh biomass (section 5).

## 5. Impact of pasteurising spinach juice on the composition and physical nature of derived dry powders that contain chloroplasts released from plant cells

We have observed that a significant percentage of galactolipids (the major lipids in chloroplasts) are hydrolysed over time in spinach juice from fresh leaves,^80^ so it is best not to leave juice from fresh spinach too long before heat treatment and/or CRF preparation. Lipolysis often favours fatty acid oxidation; free polyunsaturated fatty acids are the most efficient substrates for lipoxygenases (LOX),^111^ although these enzymes can also act on esterified fatty acids.^112–115^ Heat treatments like blanching and pasteurisation have the double advantage of inactivating both lipolytic enzymes and oxygenases naturally present in plant and algal cells. When applied to the model microalga *Chlamydomonas reinhardtii*, blanching impaired both endogenous lipolysis of galactolipids and the production of oxidation products derived from polyunsaturated fatty acids.^116^

**Fig. 14:**
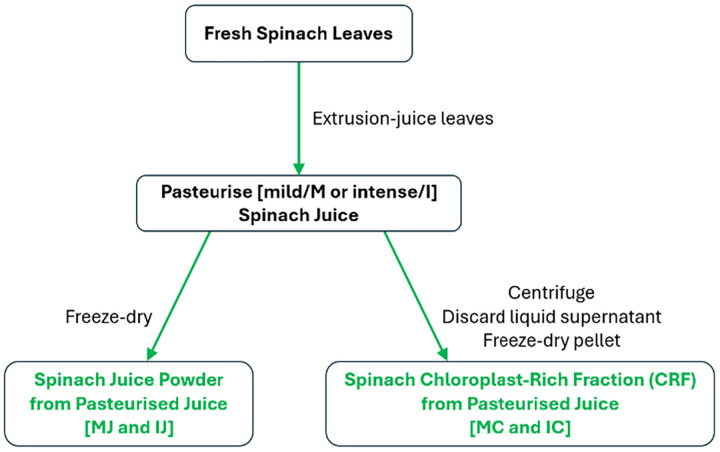
Pasteurising spinach leaf juice prior to preparing juice powder or CRF.

Fresh spinach leaves were juiced then pasteurised at 70 °C for 15 seconds (mild/M), or at 90 °C for 5 min (intense/I) (Fig. 14). Peroxidase (POD) activity in the juice was reduced by over 95% after either mild or intense heat treatment. To prepare juice powder, freeze-drying was used instead of spray-drying due to the small volume of juices that was heat-treated. The CRF was prepared as before, by freeze-drying the pellet generated after centrifugation of the filtered juice.

### 5.1. Impact of pasteurising spinach juice on the composition of derived dry powders that contain chloroplasts released from plant cells

Pasteurised spinach juice was not only freeze dried directly but also centrifuged to form pellets that were then freeze-dried (spinach leaf CRF from pasteurised juice: MC and IC in Table 6). There appeared to be temperature-dependent coagulation, with the average particle size in the juice increasing from 4.4 µm in the fresh juice, to 40.6 and 103.0 µm in the mild (M) and intense (I) pasteurised spinach juices, respectively. Approximately 20% of green leaf lipid and protein, respectively, is located outside the chloroplast. One would therefore anticipate that soluble cytoplasmic proteins would coagulate on heating and, therefore, precipitate after centrifugation, along with organelles such as chloroplasts, instead of remaining in the supernatant phase (this may be beneficial if the objective is to increase protein concentration, but antinutrients may get caught up in this precipitate). Indeed, it appears that the coagulation phenomenon caused a measurable amount of protein from outside the chloroplast to precipitate with the pellet that forms the CRF during centrifugation; this was deduced from the relative increase (about 16%) in protein and the relative decrease (about 30%) in total lipid in the CRF derived from both M and I heat-treated juice (compare FC with MC and IC in Table 6). Ash content does not appear to be affected by heat treatment and is relatively consistent across dried juice and CRF samples (approximately 19% and 7.5%, respectively). Heavy metals are not generally present in spinach leaf material, so ash content should not be an issue.

**Table 6:** Effect of juice pasteurisation on the macronutrient composition of spinach juice powder and of CRF

/%DW	FJ	FC	MJ	MC	IJ	IC
Proteins	39.6 ± 0.4^de^	50.6 ± 0.5^c^	41.2 ± 0.1^d^	58.6 ± 0.2^a^	38.5 ± 0.9^e^	54.9 ± 1.0^b^
Lipids	11.8 ± 0.4^d^	28.3 ± 0.4^a^	11.5 ± 0.5^d^	20.3 ± 0.7^b^	10.7 ± 0.5^d^	18.1 ± 0.3^c^
Ash	20.8 ± 0.2^a^	7.2 ± 0.1^d^	18.2 ± 0.5^b^	7.1 ± 0.1^d^	18.9 ± 0.3^b^	7.7 ± 0.1^d^
Carbohydrates	27.8	13.9	29.1	14.0	31.9	19.3

To appreciate the effect of juice pasteurisation on lipophilic micronutrients, we measured chlorophyll, lutein, β-carotene, α-tocopherol and α-linolenic acid in both juice powder and CRF.^82^ On a total dry weight basis, pasteurisation, both mild and intense, did not greatly affect the concentration of micronutrients in juice powder, but chlorophyll declined as heat increased, with up to 45% being lost after intense pasteurisation. On a dry mass basis, the concentration of micronutrients in CRF material, unlike the juice powder, was affected by heat, the reduction increasing with increasing intensity of pasteurisation. This can be explained by an increase in extraneous proteins, effectively diluting the concentration of micronutrients. This pasteurisation-induced decline in the concentration of micronutrients in CRF samples is less apparent if the results are expressed relative to total lipid; this is not the case for chlorophyll concentration, where a pasteurisation-induced decline is more apparent. This is consistent with the fact that chlorophyll pigments are highly sensitive to changes in temperature and humidity, which can significantly damage the photosynthetic machinery in plants.^117^ This loss of chlorophyll and retention of carotenoids and α-tocopherol was also observed on heating PVH juice at 90 °C for 3 min, with a 20 min lead up time.^118^

### 5.2. Impact of pasteurising spinach juice on the physical properties of derived dry powders that contain chloroplasts released from plant cells

After pasteurisation and freeze-drying, the dried spinach juice and CRF preparations were ground to a fine powder with a mortar and pestle and then sieved using a 250 μm mesh. After these processing steps, the changes in particle size were noted (data shown in Table 7). The mean particle size (*D*_4,3_) of dried spinach juice was increased from 43.5 μm to 69.5 μm and 101.4 μm after mild and intense pasteurisation, respectively, and the dried CRF mean particle size increased in a temperature-dependent fashion from 81.9 μm to 182.1 μm and 167.5 μm, respectively. Moreover, the span of dried juice and CRF particles decreased after both pasteurisation conditions.

**Table 7:** Particle size of freeze-dried spinach juice and CRF powders derived from fresh or heat-treated juice

/μm	FJ	FC	MJ	MC	IJ	IC
*D*_4,3_	43.48 ± 2.00^d^	81.91 ± 1.91^c^	69.53 ± 3.41^c^	182.11 ± 9.94^a^	101.38 ± 4.97^b^	167.52 ± 7.50^a^
*d*_10_	4.18 ± 0.27^d^	9.87 ± 1.03^c^	13.30 ± 1.79^c^	54.07 ± 4.00^a^	15.23 ± 0.64^c^	38.13 ± 1.90^b^
*d*_50_	29.36 ± 1.77^d^	65.85 ± 2.43^c^	68.09 ± 3.45^c^	175.29 ± 6.58^a^	91.83 ± 6.29^b^	165.76 ± 2.10^a^
*d*_90_	109.80 ± 4.77^c^	178.05 ± 3.22^b^	126.42 ± 6.48^c^	305.08 ± 14.57^a^	202.07 ± 7.82^b^	291.09 ± 14.99^a^
Span	3.60 ± 0.07^a^	2.56 ± 0.08^b^	1.66 ± 0.08^d^	1.43 ± 0.02^e^	2.04 ± 0.07^c^	1.53 ± 0.08^de^

**Fig. 15:**
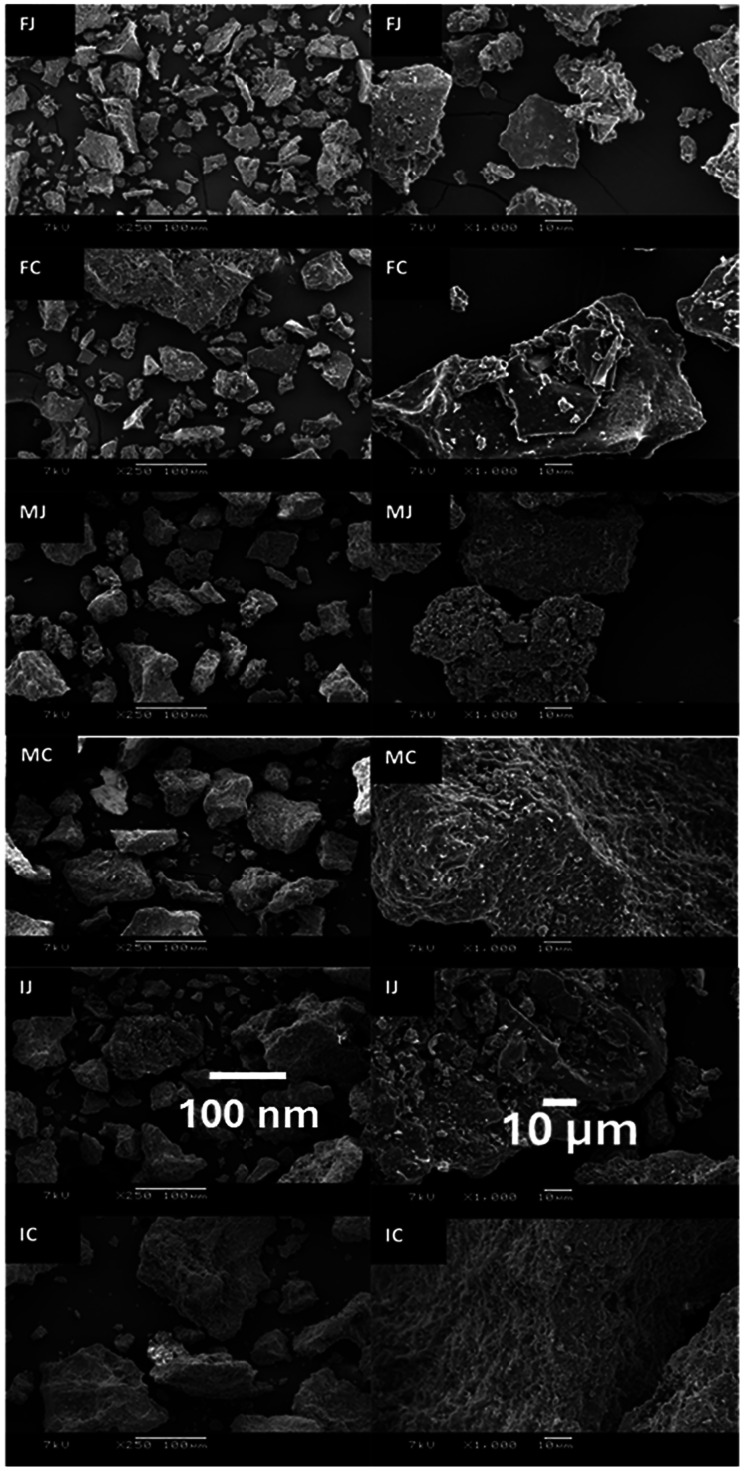
Scanning electron microscope images of powders derived from fresh or heat-treated spinach juice or the chloroplast-rich pellet derived from fresh or heat-treated spinach juice at ×250 and ×1000 magnification. Abbreviations: FJ fresh freeze-dried juice; FC fresh freeze-dried CRF; MJ mild pasteurised freeze-dried juice; MC mild pasteurised freeze-dried CRF; IJ intense pasteurised freeze-dried juice; IC intense pasteurised freeze-dried CRF. (Images from Chi, 2023.^82^)

The morphology of freeze-dried spinach juice and CRF samples was examined using a SEM at 250× and 1000× magnification (Fig. 15); the pasteurisation conditions resulted in powders with different particle morphologies. The spinach powder samples for these investigations were all obtained by freeze-drying and thus exhibited an irregular and broken glass structure, the typical structure characteristic of freeze-dried products.^119^ The size of both juice and CRF particles increased with increasing pasteurisation temperature. Also, compared with spinach juice powder, pelleting from juice to produce CRF resulted in larger particles for all treatments (Table 7). The increased size of particles observed after pasteurisation can be explained by the aggregation of proteins in spinach juice during heating.

For all treatments (fresh or heated), higher moisture content was observed in dried spinach juice (7.7–9.3%) compared with CRF materials (3.8–4.7%). For all samples shown in Table 8, the moisture content observed in this study was lower than 10% and the water activity was around 0.3. This should promote the powders’ microbiological safety and stability during long-term storage.^120^

**Table 8:** Some physical properties of freeze-dried spinach juice and CRF powders derived from fresh or heat-treated juice

Properties	FJ	FC	MJ	MC	IJ	IC
Moisture content/% wet mass	7.7 ± 0.2^c^	4.7 ± 0.2^d^	9.3 ± 0.1^a^	3.8 ± 0.1^e^	8.3 ± 0.2^b^	3.8 ± 0.1^e^
Water activity	0.23 ± 0.01^c^	0.25 ± 0.00^b^	0.34 ± 0.00^a^	0.22 ± 0.01^c^	0.17 ± 0.01^e^	0.19 ± 0.01^d^
Solubility/% wet mass	68.6 ± 0.6^a^	25.3 ± 0.3^d^	41.3 ± 0.2^c^	10.1 ± 0.1^e^	47.0 ± 0.0^b^	10.2 ± 0.1^e^

The solubility of fresh, dried juice and CRF (FJ and FC) was 68.6% and 25.3%, respectively, compared with 73.9% and 42.6% for equivalent samples from a different batch of spinach shown in Table 5. This decreased from 68.6% (FJ) to 41.3–47.0% after mild and intense pasteurisation, respectively (MJ and IJ), and from 25.3% (FC) to 10.1–10.2% after mild and intense pasteurisation, respectively (MC and IC). In section 3.3, we noted that fresh/non-heated juice powder (however it was dried) was, using the method we have employed to measure this property, more soluble than chloroplast-enriched material. The increased carbohydrates and ash in juice are more soluble in the dried state, than chloroplasts. This trend for juice powders to be more soluble than chloroplast-enriched powders is seen in Table 8. Heat treating the juice caused a reduction in solubility of all the samples by 40% and 60% for the juice and CRF samples, respectively, and the trend for juice powders to be more soluble than chloroplast powders remained. Intense heat treatment did not seem to change the reduction in solubility of the powders observed after mild pasteurisation of the parent juice. Protein denaturation/coagulation caused by thermal treatment likely contributes to solubility, as denatured proteins (likely co-precipitated with CRF) tend to be less soluble than native forms of globular proteins.

The review so far has outlined the methods we have developed to prepare cell-wall free and dry preparations of chloroplasts, the physical nature/material properties of these powders, and their composition. The nutritional credentials of chloroplasts are compelling (Table 3); the next section outlines work we have carried out to establish how easily these nutrients are released during digestion.

## 6. Chloroplast/CRF material as a natural source of bioaccessible multiple nutrients

*In vitro* digestion can be used to measure the bioaccessibility of nutrients within vegetables and fruits.^121–125^ The bioaccessibility and subsequent bioavailability (uptake of nutrients into the body and delivery to tissues) of lipophilic nutrients in chloroplasts is hindered by the cell wall and is also affected by their tight association with the protein matrix of thylakoid membranes.^12^ Galactolipids (enriched in the omega-3 fatty acid α-linolenic acid) are a major part of thylakoid membranes, so their digestion is also likely to affect the rate of release of nutrients into the micellar phase in the small intestine (bioaccessibility). By liberating chloroplasts from plant cells (CRF), we removed the cell wall barrier to digestive enzymes and established the impact of the intra-organelle matrix on the bioaccessibility of chloroplast-located lipophilic nutrients (*in vitro* digestion study^118,125^).

**Fig. 16:**
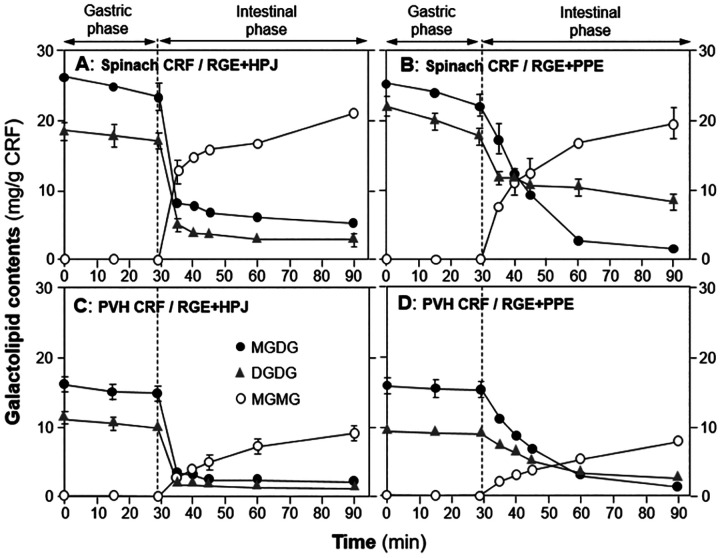
Hydrolysis of galactolipids during two-step static digestion of CRFs. CRFs from blanched spinach leaves were digested using a combination of RGE (rabbit gastric extract) and HPJ (human pancreatic juice) as sources of gastric and pancreatic enzymes respectively (panel A) or a combination of RGE and PPE (porcine pancreatic extract) as an alternative source of pancreatic enzymes (panel B). Similarly, CRF from steam sterilised PVH were digested using a combination of RGE and HPJ (panel C) or a combination of RGE and PPE (panel D). Symbols: full black circles, MGDG (monogalactosyldiacylglyerol); open circles, MGMG (monogalactosylmonoacylglyerol); grey triangles, DGDG (digalactosyldiacylglyerol). Values (mg of galactolipid per g (DW) of CRF) are means ± SD (*n* = 3). Taken from Wattanakul, 2019.^125^

**Fig. 17:**
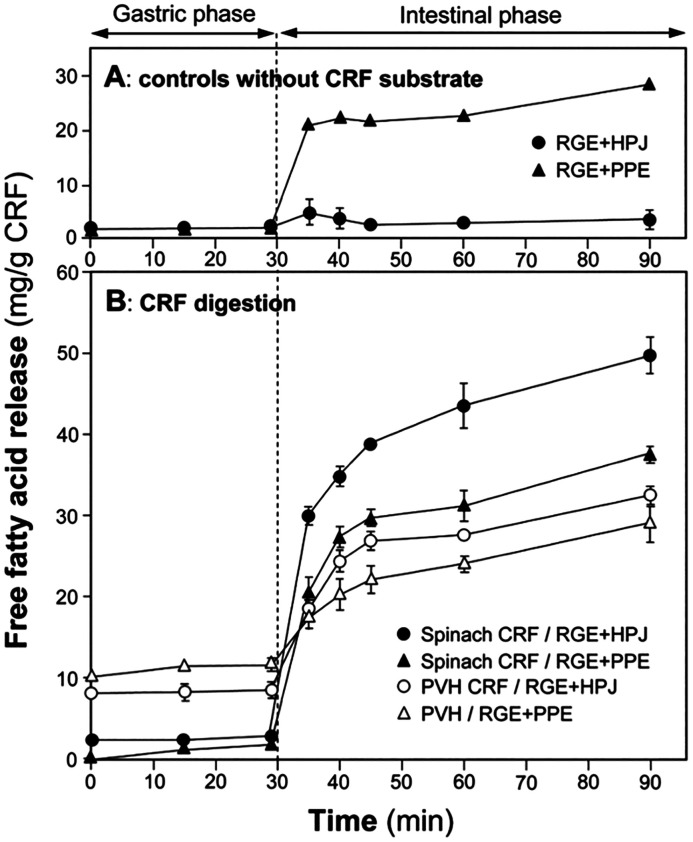
Free fatty acid released during the two-step static digestion of CRFs. (A) Blank without CRF substrate showing that some FFA can be released from PPE. (B) Digestion of CRFs from blanched spinach leaves and steam sterilised PVH, incubated with either a combination of RGE and HPJ as sources of gastric and pancreatic enzymes or a combination of RGE and PPE as an alternative source of pancreatic enzymes. Values (mg of FFA released per g (DW) of CRF) are means ± SD (*n* = 3). Figure taken from Wattanakul *et al.*, 2019.^125^

Initially, we explored the digestion of galactolipids in chloroplasts using an *in vitro* model.^125^ For these studies, CRF was generated from blanched spinach leaves, as blanching disabled endogenous galactolipase enzymes so that our experiments would reveal only the action of gastrointestinal enzymes added during the *in vitro* digestion incubation. Galactolipids in spinach leaf CRF are digested *in vitro*, mostly during the intestinal phase of digestion by pancreatic enzymes (Fig. 16 and 17). In this work, we found that human pancreatic juice (HPJ) contains enzymes that digest CRF galactolipids, presumably PLRP2 (pancreatic lipase-related protein 2) and CEH/BSSL (carboxyl ester hydrolase/bile salt simulated lipase), that are the main enzymes found in pancreatic secretions that can digest galactolipids; in addition, results suggest that HPJ is more enriched in galactolipase activity than commercial porcine pancreatic extract (PPE) commonly used for *in vitro* digestion studies, thus confirming a comparative study of these extracts with pancreatic juices.^126^ After digestion, α-linolenic acid (18:3) is the main fatty acid released from the galactolipids in CRF; this has been observed for CRF from both spinach leaves and PVH.

The physical nature of food is often overlooked as a factor determining the release of nutrients, both total release and rate of release; the latter also affects where the constituents are released in the gastrointestinal tract. The physical nature of the CRF is affected by heat treatment of the juice (section 5); therefore, changes to solubility are likely to affect the bioaccessibility of nutrients. Heat can disrupt protein-carotenoid complexes and enhance the release of both β-carotene and lutein.^124^ These carotenoids are bound to the thylakoid membranes and protect the complex against any harmful photodynamic reactions during photosynthesis.^127^ By freeing these molecules, the overall bioaccessibility of the nutritional components is enhanced.^124,128,129^ Additionally, in the presence of vegetable oil or fat, the absorption of nutrients such as carotenoids is significantly improved.^130–132^ Similarly, Nagao *et al.*^133^ observed that the presence of oil or fats during consumption helps improve the bioaccessibility of β-carotene in spinach, komatsuna, pumpkin and carrot. However, it did not improve the bioaccessibility of lutein, due to this molecule's greater hydrophilicity compared with β-carotene. Amphiphilic carotenoids (*e.g.* lutein) have a propensity to be localised at the oil/aqueous phase interface, and so they can spontaneously create micelles in the aqueous phase of the intestinal lumen. On the other hand, hydrophobic carotenoids (*e.g.* β-carotene) do not partition into the aqueous phase but migrate into mixed micelles that can deliver them to the intestinal epithelial cells.^134^

**Fig. 18:**
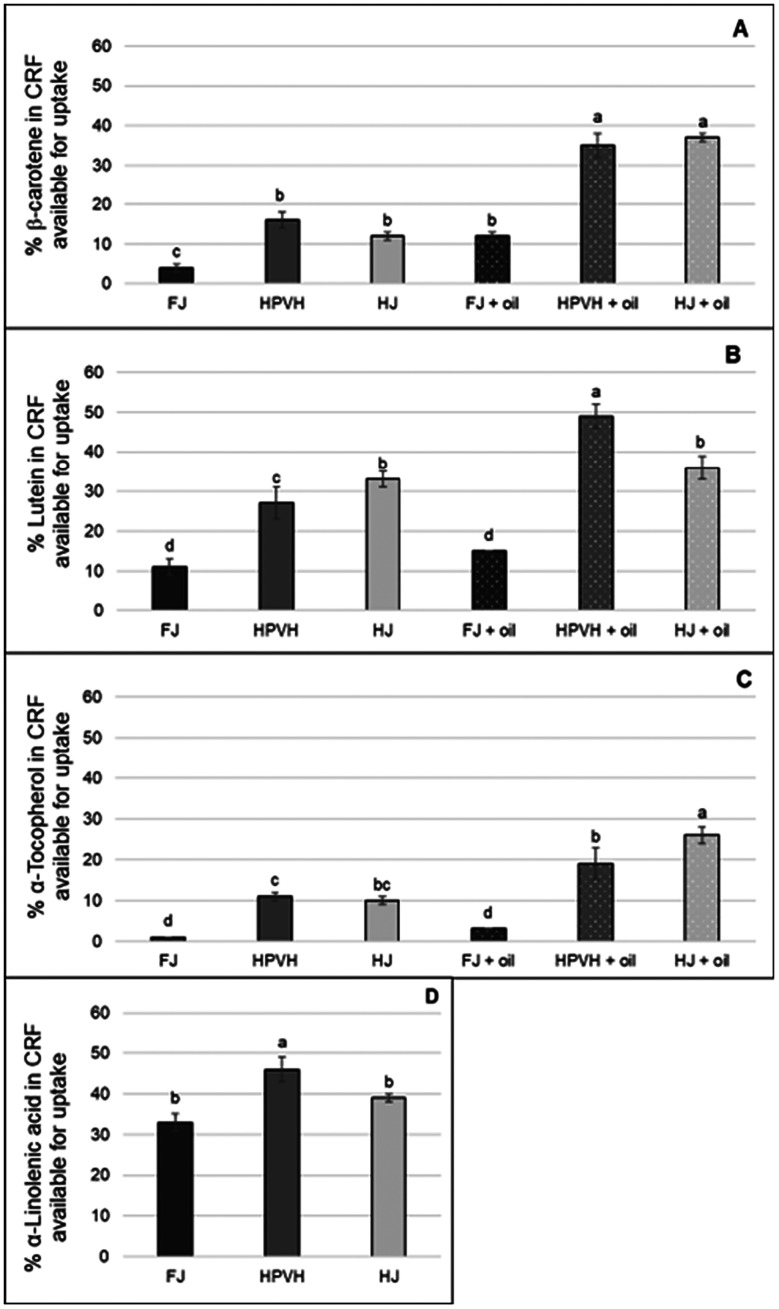
Effect of heat treatment and the presence of oil on the nutrients in CRF from PVH available for uptake after *in vitro* digestion. (A) β-Carotene available for uptake after *in vitro* digestion (B) lutein (C) α-tocopherol (D) α-linolenic acid; FJ: fresh juice; HPVH: heated PVH; HJ: heated juice; data are presented as a mean ± SD of 3 separate *in vitro* digestion incubations and analysed using *post hoc* analysis of variance (ANOVA) and according to a Tukey test with statistical significance at *p* < 0.05, *a* > *b* (‘*a*’ in the bar graph gives the highest mean value when compared with other groups, followed by *b*, *c* and *d*, respectively). ‘% available for uptake’ in this study was established by measuring nutrients in the micellar phase at the end of digestion and dividing that by the total present at the start of the incubation. Figure taken from Wattanakul *et al.*, 2022.^118^

Our group^118^ has measured the impact of heat-treatment of the biomass and juice, and the presence of oil, on the release of lipophilic nutrients from the chloroplast matrix into the micellar phase of the digestate in an *in vitro* model of human digestion. CRF was derived from fresh PVH (FJ), heat-treated PVH (HPVH – biomass heated to 100 °C, over 5 min, then held for 4 min), or heat-treated PVH-juice (HJ – PVH juice gradually heated to 90 °C, over 20 min, then held for 3 min). The impact of oil on the ‘percentage of nutrient available for uptake’ (a measure of bioaccessibility) was also measured for β-carotene, lutein, and α-tocopherol (Fig. 18). The static digestion model used in this study did not fully disperse the CRF powder from fresh biomass; this limitation was less pronounced when CRF from heat-treated biomass or juice was used. The addition of oil to the incubation mixture increased the bioaccessibility of β-carotene and α-tocopherol (data not available for α-linolenic) and improved the dispersion of CRF made from fresh PVH. Heating the biomass or juice appears to promote the liberation of more tightly bound lipophilic micronutrients (in this case, β-carotene). Bioaccessibility measurements in this study suggest that significant amounts of lipophilic nutrients in CRF are released and made available for uptake into the body. It appears from this work that liberating chloroplasts from their cell wall confines (reducing their degree of bioencapsulation) and concentrating them into a chloroplast-rich fraction (CRF) is an excellent way to prepare a natural, multi-nutrient material for humans.

The relatively high ‘available for uptake’ value for α-linolenic acid compared with the other lipophilic nutrients is notable (Fig. 18). We assume this is in the form of a free fatty acid, but it is possible that small segments (under 0.22 µm in diameter) of thylakoid membrane, or intact galactolipids, are released from the CRF material into the aqueous phase. It is intriguing to consider the probable direct role that free α-linolenic acid has in forming micelles and so assisting the micellisation of carotenoids from the same organelle. Further work is required to fully characterise the nature of α-linolenic acid in the micellar phase after the digestion of CRF material and to establish to what extent this fatty acid, or indeed the parent galactolipids, helps to micellise lipophilic micronutrients.

Recent work by Sahaka M. and co-workers^135^ revealed that it is possible to monitor the action of the galactolipase enzyme PLRP2 using infrared spectroscopy. Intact chloroplasts, in the form of CRF, were tested alongside pure galactolipid mixed micelles; the results showed that even in the absence of bile salts, PLRP2 is able to release almost all of the fatty acids from chloroplast membrane galactolipids. Moreover, two other studies with whole plant leaves and microalgae revealed that PLRP2 can efficiently hydrolyse galactolipids from thylakoid membranes even in the presence of the plant or microalgal cell wall.^116,136^ Therefore, digestive enzymes, as large as PLRP2 (50 kDa), can cross the plant cell wall to access first the cytoplasmic membrane and then organelle membranes. These findings suggest the enzyme penetration *via* ‘pores’ in the polymer matrix of the cell walls, including plasmodesmata. The size range of cell wall pores of different plants has been estimated to be between 3.5 to 5.2 nm ^137^ to accommodate the large globular catalytic domain of pancreatic lipase (5 nm diameter; see 3D structure in PDB 1N8S). PLRP2 can also digest plant membrane phospholipids thanks to its phospholipase A1 activity^138^ and this activity may be coupled to that of pancreatic phospholipase A2. Once the chloroplast outer and inner membranes are digested, PLRP2 can act on the galactolipids from the thylakoid membranes.^135^ There is therefore no need for mechanical or thermal cell disruption for the digestion of chloroplast lipids to occur, although it will be more rapid in the absence of the cell wall barrier.

To better appreciate the accessibility of digestive enzymes to chloroplast lipids and their interaction with lipid substrates we must consider the microstructure of chloroplasts (refer to section 1). The prevalence of glycerolipids outside of the protein complexes (90%) suggests that they are relatively accessible to digestive enzymes such as PLRP2. Indeed Boekema and co-workers^139^ estimated that the average lipid bilayer region between the protein complexes is 130 nm^2^, or 200–300 lipid molecules in one leaflet of the bilayer; given the dimensions of PLRP2 (approximately 60 × 60 × 80 Å), one could estimate that at least 3 PLRP2 enzyme molecules could cover 130 nm^2^ of glycerolipid molecules. ‘True’ lipases such as gastric lipase and pancreatic lipase sit at the surface of oil droplets and hydrolyse neutral triacylglycerol molecules in the core of the droplet, often with the help of a coenzyme such as colipase. Other lipolytic enzymes, such as pancreatic phospholipase A2, PLRP2 and CEH/BSSL, are functional at the interface of mixed micelles where their substrate, polar lipids, are directly exposed to these enzymes. It is worth noting that researchers have used lipolytic enzymes such as phospholipase A2 (PLA2 – approximate dimension of 50 × 50 × 50 Å) to explore the physical nature of glycerolipids (polar lipids) in thylakoid membranes;^8^ this demonstrates the ease of direct access of lipolytic enzymes to the glycerolipids in the thylakoid membrane.

Although our research into the digestion of chloroplast has focussed on the hydrolysis of galactolipids and the release of lipophilic nutrients, it is worth commenting on the digestion of chloroplast proteins. Overall, there are between 2000 and 3000 different proteins in chloroplasts, by mass approximately 50% are in the stroma (the majority being RuBisCO), 40% are part of the thylakoid membrane, and 10% are in the lumen. Just as lipolytic enzymes have been used as a tool to understand the nature of glycerolipids in the thylakoid membrane, proteolytic enzymes such as trypsin (63 × 63 × 70 Å) and proteinase K (70 × 70 × 110 Å) have been used to digest specific endogenous enzymes in the lumen of thylakoids, causing a change in the phase signal of lipids suggesting the importance of enzyme–lipid interactions, for example the possible role of MGDG in the H_II_ phase for the action of lipocalin proteins in the lumen such as violaxanthin de-epoxidase (VDE). Studies such as these suggest that proteolytic enzymes can not only access the domains of protein complexes exposed to the stromal phase or the partition region between thylakoid bilayers, but also the proteins within the less accessible lumen region. This notwithstanding, the transmembrane nature of most thylakoid proteins is likely to slow down their digestion compared with soluble stromal proteins. It seems likely that during mammalian digestion of chloroplasts, the galactolipids are readily digested, perhaps followed by the release of pigments that are tightly associated with the thylakoid proteins. At the microstructure scale, intact thylakoid membranes from chloroplasts may also emulsify oils during digestion by a process of Pickering stabilisation (see section 7), creating a situation where the proteins and lipids in the thylakoid initially coat and protect the oil from digestion but are themselves subject to gradual hydrolysis.

In addition to *in vitro* digestibility studies, we have also measured the bioavailability of nutrients from spinach CRF material when added to the diet of zebrafish to replace some of the fishmeal.^140^ Chloroplast marker lipids such as α-linolenic, hexadecatrienoic acid (16:3 n-3), and carotenoids (*e.g.* lutein) were taken up into the fish, lutein being particularly concentrated in the eggs of females. Vitamin A also increased in the fish, indicating the uptake of chloroplastidial β-carotene (provitamin A) into the body of the fish, and its subsequent conversion into retinol. Besides showing the digestibility and bioaccessibility of CRF materials, this study was also one of the first to indirectly show the effective digestion of galactolipids and absorption of their fatty acid in an animal species. Indeed, the presence of 16:3 fatty acid in the zebrafish lipids can be considered as a trophic marker of the transfer of fatty acids from plant galactolipids to the fish.^141^

## 7. Surface-active properties of CRF and chloroplast membrane material (CMM)

### 7.1. Surface-active behaviour of CRF during the digestion of oils and fats

**Fig. 19:**
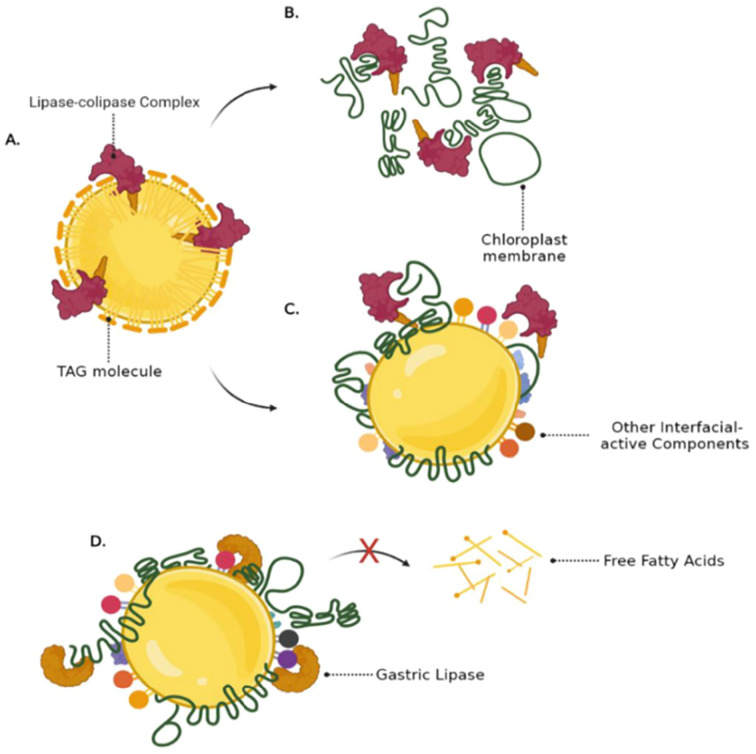
Schematic representation of chloroplast membranes interfering with the oil digestion (*i.e.*, TAG hydrolysis). (A) The lipase–colipase complex binds to the fat droplet in the absence of chloroplast membranes and begins the hydrolysis of the fat molecule. (B) The lipase–colipase complex gets adsorbed to the free floating chloroplast membrane system and shields the binding site of the enzyme. (C) The chloroplast membrane system (and its interfacial-active components) adsorbs at the oil-and-water interface and interferes with the binding of the lipase–colipase complex to its substrate. (D) The chloroplast membrane system (and its interfacial-active components) interferes with the binding of gastric lipase to the substrates during the gastric phase; a direct reduction in free fatty acid (FFA) production would have a significant impact on overall pancreatic lipase activity.^146–149^ Image taken from Sucharit, 2022.^81^

Our digestion work focused on the breakdown of whole chloroplasts and the release of nutrients. We demonstrated that galactolipids in CRF material are digested by human pancreatic juice containing PLRP2 and CEH/BSSL.^125^ Pure thylakoid membranes have a high affinity for oil and water interfaces^77,142,143^ and it has been shown that chloroplast membranes can slow down TAG (triacylglycerol) digestion by blocking the binding of the lipase–colipase complex and bile salts to the oil-and-water interface^18,144,145^ (Fig. 19).

This raises the question as to whether CMM (chloroplast membrane material, rich in thylakoid membranes) is released from intact chloroplasts during digestion and remains intact/undigested long enough in the gastrointestinal tract to retain its ability to cover oil droplets and so, potentially, reduce the rate of triacylglycerol (oil and fat) digestion.

**Fig. 20:**
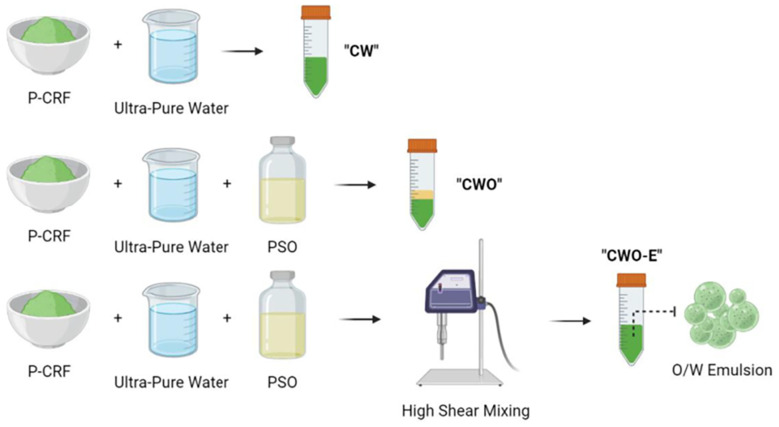
Preparation of CW, CWO, and CWO-E. CW: 0.25 g of P-CRF (equivalent to CRF) was mixed with 4.75 g of ultra-pure water (top). CWO: 0.25 g of P-CRF was mixed with 3.75 g of ultra-pure water and 1 g of purified sunflower oil (PSO) (centre). CWO-E: was prepared the same way as CWO with an additional high shear homogenisation step (20 500 rpm, 2 min) (bottom). All samples were stored on ice prior to digestion experiment. Image taken from Sucharit, 2022.^81^

To address this question, within an *in vitro* model of human digestion, we combined CRF (rich in galactolipids) and oil (rich in triacylglycerol/TAG molecules), either as discrete materials, CRF/water/oil mix (CWO), or as an emulsion, homogenised CRF/water/oil mix (CWO-E) (Fig. 20). After exposure of these preparations to lipolytic enzymes, RGE (rabbit gastric extract) in the gastric phase, and PPE (porcine pancreatic extract) in the intestinal phase, products of galactolipid and TAG hydrolysis were used as a measure of their digestion. Overall, the results^81^ suggest that, in the presence of oil (CWO), the galactolipids in CRF material become more resistant to lipolysis. Pre-emulsification, to optimise the coverage of chloroplast membranes at the oil-and-water interface (CWO-E), further protects galactolipids from hydrolysis. Not only does oil slow down the digestion of galactolipids in CRF, but the presence of CRF reduces the rate of the digestion of TAGs. As was seen when measuring galactolipid digestion, TAG digestion is slower in the CWO-E emulsion than when CRF and oil are added as separate entities (CWO). One possible explanation is the partitioning of lipases when separate entities are used: the pancreatic lipase binds preferentially to oil droplets, while PLRP2 binds to chloroplast membranes, a property recently shown with CRF^135^ as well as with heterogenous plant lipid model membrane.^138^ With the CWO-E emulsion, only one type of interface is available, where the presence of CMM might impair pancreatic lipase penetration/access to TAG, and PLRP2 activity on galactolipids could be reduced. Our work used CRF material, and the extent of chloroplasts breaking and therefore releasing CMM during the experiments was not established. It may therefore be possible that intact chloroplasts, or at least partially disrupted/digested chloroplasts, as well as CMM, possess the ability to retard fat digestion and so induce satiety, and that this property could be maximised through a ‘pre-emulsification’ step.

### 7.2. Surface-active behaviour of CRF material in oil-in-water emulsions and in a fat-continuous model food

**Fig. 21:**
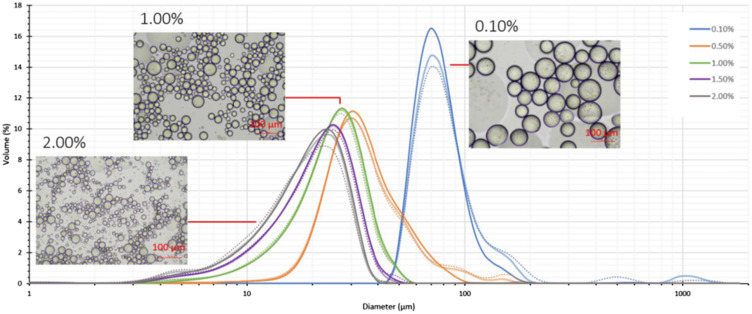
Droplet size distribution (DSD – measured using laser light scattering) of P-CRF stabilised o/w emulsion over storage at 4 °C. Solid lines (day 1). Faded colour lines (day 2). Dotted Lines (day 3). Micrographs were obtained using the transmitted light technique at ×200 total magnification. Scale bar indicates 100 μm. Image taken from Sucharit, 2022.^81^

The majority of proteins and lipids that constitute the thylakoid membrane are amphiphilic in nature, therefore there is good reason to test the surface-active properties of chloroplasts and their derivatives. Oil-in-water (o/w) emulsions were prepared using spinach P-CRF material (equivalent to the CRF material already described), oil and water.^81^ The o/w droplet size distributions (DSD) shown in Fig. 21 indicate that the greater the amount of CRF material added, the smaller the diameter of the emulsion droplets. The CRF material can stabilise an o/w emulsion for several days at 4 °C. This is consistent with Rayner and co-workers^142^ and with Tenorio and co-workers;^77^ both research teams created o/w emulsions, using thylakoid membranes prepared in slightly different ways; these emulsions remained stable for over seven days at 4 °C. Tenorio and co-worker^77^ provided strong evidence that thylakoid fragments stabilised oil-in-water droplets through a Pickering (particle) mechanism, this seems likely given that 1. 70% of the thylakoid membrane is composed of the four protein complexes, each rich in transmembrane proteins that would not easily or quickly disentangle and align their complex hydrophobic core to the oil surface, and 2. The bilayer regions between these complexes are rich in glycerolipids that do not display the geometry that promotes a positively curved lipid monolayer that is required to stabilise oil-in-water emulsions. The precise mechanism by which our CRF preparation stabilises oil/water interfaces is not known. Given that stromal globular proteins such as RuBisCO are present in our CRF preparations, the stability of oil droplets in a continuous aqueous phase could be *via* Pickering (particle) stabilisation from the intact thylakoid membrane fragments, or through the unfolding of globular proteins at the oil/water interface, or both to varying degrees over time. Given the range of possible mechanisms, the kinetic dimension of interfacial behaviour of chloroplasts/CRF should not be overlooked.

We have extracted lipid from spinach chloroplasts and tested the ability of this extract to influence the flow of material in an oil-continuous phase.^150^ We have also extracted lipid from whole spinach leaves and showed a yield stress reduction in a concentrated sugar-in-oil suspension.^151^ Since spinach lipid contains a substantial amount of MGDG and DGDG, the flow enhancing ability observed may be a result of galactolipids,^152,153^ but the other glycerolipids should not be overlooked; given the geometry of the glycerolipids in chloroplasts (see Fig. 5) it seems more likely that DGDG, SQDG and PG, rather than the most abundant MGDG, would be responsible for this physical effect. We also probed the surface-active properties of the intact, multicomponent thylakoid/chloroplast membrane material; enhanced surface-active properties would justify the commercial release of thylakoid membranes from chloroplasts, offering an increasing range of applications in addition to the nutritional value of intact chloroplasts. This would also allow the use of leaf material as a surface-active ingredient without organic solvent extraction. A model chocolate system (a 65% (w/w) sugar-in-oil suspension) was developed to assess the ability of natural surfactants as a potential replacement for the synthetic emulsifier polyglycerol polyricinoleate (PGPR) that is used to reduce the yield stress of molten chocolate in some formulations.^154^ Using this model of a fat-continuous food system, we demonstrated that spinach CMM (chloroplast membrane material), with varying degrees of membrane integrity, can reduce yield stress, flow point, and apparent viscosity values in a fat-continuous model chocolate system.^155^ These results suggest that natural CMM could replace synthetic PGPR (polyglycerol polyricinoleate) as a flow enhancer in chocolate formulations.

**Fig. 22:**
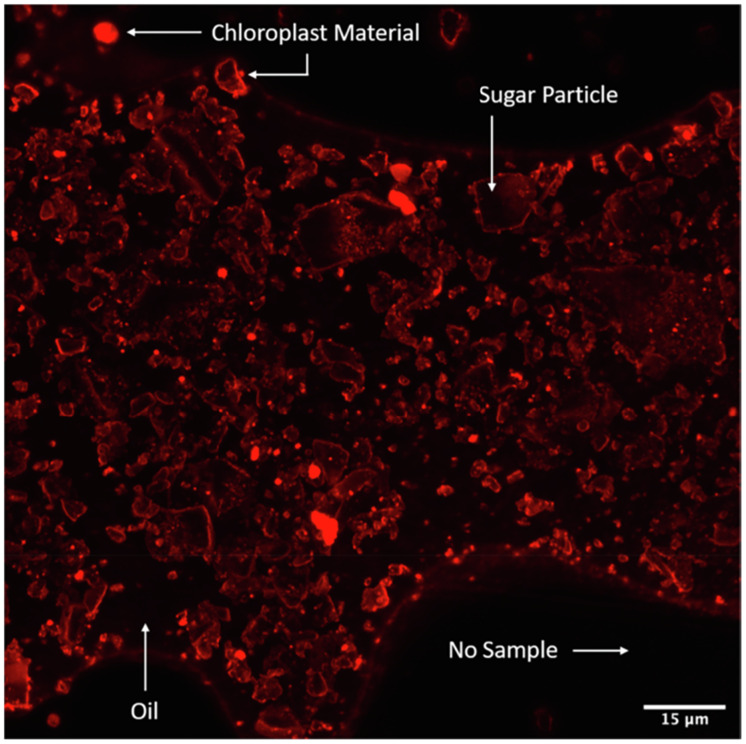
Maximum intensity projection confocal micrographs of 1.0% S-CRF (rhodamine B-stained chloroplast-rich fraction) in 50% sugar in oil (s/o) suspension at ×63 objective lens (*Z* stacks = 20). Under a 561 nm lamp, structures that appear in red indicate the protein-stained chloroplast material (rhodamine B, *λ*_ext_: 553 nm, *λ*_emi_: 627 nm). Black region is area with no sample, while the lighter red region indicates the oil phase. Scale bar represents 15 μm. Image taken from Sucharit *et al.*, 2023.^155^

Confocal micrographs from this work (Fig. 22) provide an insight into the amphiphilic nature of CRF, as it arranges itself at the sugar and oil interface. Through thermodynamics, thylakoid membranes stabilise sugar and oil interfaces and enhance the flow in an oil-based system. These surface-active properties are likely to vary, depending on how the CRF/CMM is prepared. Specific methods to prepare unravelled thylakoid membranes could be developed through an understanding of the forces that hold stacks of thylakoid membranes together. Due to the generation and movement of protons in sunlight, the pH of the stromal region of chloroplasts is approximately 8.0 and that of the lumen is approximately 5.0. In the dark, the pH in these regions tend to move towards 7.0. In both the light and the dark there is a net negative charge on the surface of thylakoid bilayers due to the presence of acidic lipids, PG and SQDG, and exposed carboxyl groups of amino acids associated with membrane–protein complexes; the degree of negative charge tends to increase in the light. The laminated structure of thylakoid membranes is therefore retained by the presence of counterions such as Mg^2+^ which negate the tendency for thylakoid membranes to separate due to electrostatic repulsion. Chelators of divalent cations, and gentle heat 35–45 °C^156^ could be used to de-stack/unravel thylakoid membranes, thus tailoring its surface activity and maximising its surface coverage. It is also worth noting the broad processing strategies that can be applied to tailor the functionality of plant-derived protein-based emulsifiers.^157^

## 8. Analogies between CRF and chlorophyll-paste used in cooking – potential applications in gastronomy

CRF preparation is reminiscent of the traditional preparation of chlorophyll-juice or -paste in cooking recipes.^158–160^ Chlorophyll preparation usually involves blending green leaves (spinach, parsley, cress) in water for a few minutes. The bright green liquid obtained is then passed through a fine sieve, heated at low temperature (around 70 °C) with constant and slow stirring until green particles rise to the surface. The green scum that forms is collected with a ladle and poured through a muslin-lined sieve placed on ice. Once cooled and drained, the green chlorophyll-paste remaining on the muslin is scraped and can be either used for cooking or kept in the fridge for up to 1 week. The short heating process stabilises the green colour of the paste, which can then be used for colouring sauces, soups, dressings, sweets and pastries. Thus, chlorophyll-paste can be added to pasta dough, scrambled eggs, risotto, soup, butter and mayonnaise to achieve a bright green colour. Chefs use chlorophyll to create visually striking gourmet dishes. This natural pigment not only enhances the visual appeal of the dishes but also adds a layer of fresh, earthy flavour that complements other ingredients. Molecular gastronomy has also adopted chlorophyll to create unique textures and presentations using techniques of encapsulation with agar gel (green pearls and edible films).

The use of chlorophyll in the food industry is expanding rapidly, with new and innovative applications emerging in confectionery and haute cuisine. As consumers are seeking out natural and health-boosting ingredients, the incorporation of CRF in various food products is likely to offer visual appeal, and to enhance flavour, in addition to its nutritional contents and health benefits. There are, however, some stability challenges regarding chlorophyll. The effects of pH on chlorophyll degradation and colour loss were observed in blanched green peas. The rate constants of green colour loss and chlorophyll degradation decreased with increasing pH, indicating that the green colour could be retained at higher pH conditions.^161,162^ The chloroplast-rich fraction is likely to be seen as a novel food in the UK and EU, as it is an extract or fraction of an existing food (as exemplified by an application for defatted chia powder^163^); however, in the US it could be treated as a concentrated botanical fraction, requiring a New Dietary Ingredient notification with evidence of safety before marketing. More information should be sought from appropriate regulatory bodies before using it as an ingredient in food or a supplement.

## 9. Summary and conclusion

Chloroplasts are hyperabundant in the biosphere and offer (intact or fractionated) a renewable source of ingredients for a range of applications. Simple physical processes can be used to break open cells and to separate chloroplasts from the parent green biomass. Our studies testing the impact of heating and drying regimes on the physical and nutritional quality of a range of chloroplast-rich powders, suggest that there is some versatility in the processes selected, depending on the final application. What is apparent is the need to process the biomass in as fresh a state as possible. The nutritional profile of chloroplasts marks them out as notable, natural food supplements or food ingredients; these unique, complex, single particles are a rich source of pro-vitamin A, vitamin C, vitamin E, lutein, vitamin K1, α-linolenic acid (an essential fatty acid), iron and manganese. *In vitro* digestion studies using human pancreatic juice have shown that galactolipids in intact chloroplasts are digested and that the lipophilic nutrients are released (made bioaccessible). Results from fish feeding trials support this deduction and, furthermore, indicate the bioavailability (as well as the bioaccessibility) of these lipophilic nutrients.

We have explored the surface-active properties of the thylakoid-rich chloroplast membrane material (CMM), showing that this multicomponent membrane material is able to improve the flow properties of a model chocolate formulation. The drive in the food industry towards natural and minimally processed ingredients justifies further work exploring the functional, as well as the nutritional, properties of chloroplasts. The surface-active properties of CMM could also be deployed in several pharmaceutical applications, such as the delivery of hydrophobic bioactives.

Although this review has focused on lipophilic nutrients, chloroplasts also contain the world's most abundant protein – ribulose-1,5-bisphosphate carboxylase/oxygenase (RuBisCO). This protein is rich in essential amino acids and is likely to offer a range of functional properties of interest to food material scientists developing more sustainable food formulations. RuBisCO is located in the stromal region of the chloroplast, so a case can be made not only to recover intact chloroplasts from underutilised green biomass for direct applications, but also to include an optional fractionation step to separate CMM from RuBisCO, thus increasing the range of applications of chloroplasts and their derivatives.

## 10. Future research

Chloroplasts have been overlooked in terms of their potential benefits to mankind *ex vivo*; also, given their macro and micro-nutrient composition, chloroplasts could also be a viable alternative to fish meal in the growing global aquaculture industry as suggested by our study performed with zebrafish.^140^ While the nutritional credentials of this organelle, both in terms of its composition and the bioavailability of its lipophilic nutrients, have been established, we have yet to measure the bioavailability of iron from chloroplasts. Plant sources of iron are less bioavailable than animal sources; it is possible that plant cell walls (resistant to digestion in humans) act as a barrier to the uptake of iron. With a drift towards vegan diets in some populations, a preparation of chloroplasts released from plant cells might provide a good source of iron, as well as other nutrients. One overlooked aspect in our work so far is the flavour of our chloroplast preparation: given the composition of chloroplasts, their consumption may elicit an umami sensory response similar to the sensory response on consuming matcha, a dry powder of fresh tea leaf. Informal tasting of our CRF preparation by a limited number of untrained consumers has indicated that it has a pleasant savoury flavour profile, so it is likely that chloroplast-rich powders could be used as a seasoning for food, as discussed here regarding the traditional use of chlorophyll paste in gastronomy and much like some seaweeds that are also used. Chloroplast preparations could therefore help to improve the appetite of older populations, and so improve their diet-related health.

In considering chloroplasts as a nutritionally-valuable ingredient for human consumption, we have identified potential food safety questions which require definitive answers. For example, chloroplasts contain nucleic acids such as purine that are associated with increased incidence of hyperuricemia. Plant sources of food, compared with animal sources, contain substantially lower amounts of purines.^164^ Furthermore, a longitudinal study on the association of dietary protein sources and incidence of gout highlighted that intake of purine-rich vegetables did not significantly associate with gout development.^165^ While this does not directly remove the risks associated with chloroplast-rich extracts from spinach, a vegetable that is relatively rich in purine, it highlights that it is not likely to be a greater cause for concern than non-dairy, animal sources of protein in the development of gout.^164,165^ Certainly, future implementation of chloroplast-rich extracts from green leaf biomass should explore the bioavailability of purines in comparison to other plant protein sources.

Plants have the potential to accumulate heavy metals from the environment either through their roots or directly through their leaves where they accumulate. These metals, such as cadmium, zinc and lead, tend to be stored in vacuoles as phytochelatins or metallothioneins.^166^ It is not known if these complexes would precipitate with a chloroplast pellet during centrifugation, but the relatively low molecular mass of these chelating proteins (approximately 7 kDa) suggest that they may stay in the supernatant: if this were the case, then the preparation of CRF material from green biomass would be an effective way of separating toxic heavy metals from the nutritionally-rich chloroplasts. Research into this would therefore be justified.

We have worked with pea vine haulm (PVH), the post-peavine-harvest field residue, to generate a chloroplast-rich fraction (CRF). We estimate that if all UK PVH were turned into CRF, it could provide the vitamin A and iron (if bioavailable) nutrient requirements for approximately 2 million adults for a whole year. Other post-harvest field residues could be studied in future. Future research can focus on putting underutilised biomass to use and also addressing two key questions for the primary producer/farmer:

1. If this biomass is usually ploughed back into the soil, what impact would removing a proportion of this biomass from the field have on soil health?

2. If the green biomass needs to be processed as fresh as possible, is there an on-farm solution to this challenge?

Economics will drive the adoption of this technology, so developing a process not only to release chloroplasts from plant cells, but also to create more products for sale by fractionating chloroplasts into a chloroplast membrane material (CMM) fraction and a RuBisCO-rich stromal fraction would be of real interest.

## 11. Closing comments

It is estimated that the total live biomass on Earth is 550 billion (giga) tonnes, with plants and algae providing 80% of this mass. A small proportion of this abundant photosynthetic biomass already provides food for mammals. Herbivores can extract more nutritional value from green biomass than omnivores, so the liberation of chloroplasts from plant cells could be a game-changer for feeding a growing global human population. Such chloroplast-rich preparations could be consumed directly or fed to omnivorous or carnivorous animals, such as fish, that are consumed by humans. This review sets out the work we have carried out on chloroplast-rich extracts to: optimise methods of extraction/recovery; measure their nutrition content and the bioaccessibility of the lipophilic nutrients; establish the physical properties of the dried powders; disrupt intact chloroplasts to release thylakoid membranes/chloroplast membrane materials (CMM); and measure the surface-active properties of CMM. A platform has been prepared for further research and development into chloroplasts that should lead to a wide range of applications that could address the UN Sustainable Development Goals 2, 3, and 12 (zero hunger, good health and well-being, and responsible consumption and production, respectively). As such, this is an area of research which more than warrants further intensive work and investment.

## Author contributions

David Gray: conceptualization; writing – original draft; data curation; formal analysis; funding acquisition. Poramat Sutcharit: data curation; formal analysis; writing – original draft. Jutarat Wattanakul: data curation; formal analysis. Mohamed A. Gedi: formal analysis. Ruth Price: formal analysis; data curation; writing – review & editing. Ardeshir Farmanfarmaian: methodology. Randa Darwish: data curation; formal analysis. Syamila Mansor: data curation; formal analysis. Chao Chi: data curation; formal analysis. Malgorzata Walczak: data curation; formal analysis; writing – review & editing. Rhianna Briars: data curation; formal analysis. Joshua Reid: formal analysis. Vincenzo Di Bari: formal analysis. Joanne Gould: formal analysis. Molly Muleya: data curation; formal analysis. Robert Rintoul: investigation. Lorna Mcausland: investigation. Moulay Sahaka: data curation; formal analysis. Frédéric Carrière: writing – review & editing.

## Conflicts of interest

There are no conflicts to declare.

## Abbreviations

AA: Amino acids
ATP: Adenosine triphosphate
Aw: Water activity
CAM: Crassulacean acid metabolism
CEH/BSSL: Carboxyl ester hydrolase/bile salt stimulated lipase
CHO: Carbohydrate
CMM: Chloroplast membrane material
CRF: Chloroplast-rich fraction, usually in the freeze-dried state (see FC)
CW: CRF/water mix
CWO: CRF/water/oil mix
CWO-E: Homogenised CRF/water/oil mix
DGDG: Digalactosyldiacylglycerol
DSD: Droplet size distribution
Fd: Ferredoxin
FD: Freeze-dried
FFA: Free fatty acid
FJ: Freeze-dried juice
FC: Freeze-dried CRF
FNR: Ferredoxin-NADP-reductase
GLV: Green leafy vegetables
HJ: Heated juice
HPJ: Human pancreatic juice
HPVH: Heat-treated PVH
IC: Intense pasteurised freeze-dried CRF
IEM: Inner envelope membrane
IJ: Intense pasteurised freeze-dried juice
LHC: Light harvesting complex
LOX: Lipoxygenase
MC: Mild pasteurised freeze-dried CRF
MGDG: Monogalactosyldiacylglycerol
MJ: Mild pasteurised freeze-dried juice
NADH: Reduced nicotinamide adenine dinucleotide
NPQ: Non-photochemical quenching
NRI: Nutrient reference intake
OEM: Outer envelope membrane
PG: Phosphatidylglycerol
PGPR: Polyglycerol polyricinoleate
PLRP2: Pancreatic lipase-related protein 2
POD: Peroxidase
PPE: Porcine pancreatic extract
PSI: Photosystem I
PSII: Photosystem II
PVH: Pea vine haulm
RGE: Rabbit gastric extract
RH: Relative humidity
ROS: Reactive oxygen species
RuBisCO: Ribulose-1,5-bisphosphate carboxylase/oxygenase
SD: Spray-dried
SEM: Scanning electron microscopy
SJ: Spray-dried juice
SQDG: Sulfoquinovosyldiacylglycerol
TAG: Triacylglycerol
TEM: Transmission electron microscopy
VDE: Violaxanthin de-epoxidase
WBCRF: Water-burst CRF

## Supplementary Material

FO-017-D5FO03797B-s001

## Data Availability

This review cites data that has already been published. Supplementary information (SI) is available. Composition comparison of leaves from different sources. See DOI: https://doi.org/10.1039/d5fo03797b.
