# Supplementary material for: Chloroplasts as ingredients for food: a review
Source: Food Funct. 2026 Jun 4;17(12):5333–59. doi: 10.1039/d5fo03797b (PMC13235429; doi:10.1039/d5fo03797b)
Supplement: FO-017-D5FO03797B-s001 [file FO-017-D5FO03797B-s001.pdf]

Supplementary Information (SI) for Food & Function.
This journal is © The Royal Society of Chemistry 2026

Supplementary Material: Composition comparison of leaves from different sources

A. Sample moisture content and dry weight

The average moisture content ($100mg^{-1}$) and dry weight fraction (% DW) data for leaf Chlorophyll Rich Fraction (CRF) and whole leaf materials across four plant types: spinach, kale, nettles, and grass

	**Leaf CRF (% DW)** [1] ^				**Whole Leaf Materials (% DW)** [1] *			
	Spinach	Kale	Nettles	Grass	Spinach	Kale	Nettles	Grass
Moisture Content ($100mg^{-1}$ FW)	79 ± 0.3	82 ± 0.4	70 ± 0.7	72 ± 0.5	94 ± 0.2	82 ± 0.5	77 ± 0.8	80 ± 1.2
Dry Weight (DW) Fraction ($100mg^{-1}$ FW)	21	18	30	28	6	18	23	20

B. Broad Composition

The broad composition of different samples for average protein, lipid, carbohydrate (CHO) and ash content, including the whole-leaf and chlorophyll Rich Fraction (CRF) of four plants (Spinach, Kale, Nettles and Grass) and three commercial products/ preparations

	**Leaf CRF (% DW)** [1]				**Whole Leaf Materials (% DW)** [1]				**Commercial Products (% DW)**		
	Spinach	Kale	Nettles	Grass	Spinach	Kale	Nettles	Grass	Matcha or MacchaTea [2-5]	*Chlorella vulgaris* [6-8]	Spirulina (ssp. *Arthrospira*) [9,12]
Protein	42.6 ± 0.1	44.1 ± 2.7	18.3 ± 1.1	30.2 ± 0.2	35.3 ± 4.5	36.6 ± 4.8	28.0 ± 0.3	23.1 ± 1.2	17.3 - 29.6	37.6 - 58	55-70
Lipid	36.9 ± 1.3	36.1 ± 1.7	29.8 ± 1.5	30.4 ± 2.7	19.3 ± 1.6	17.0 ± 0.6	13.4 ± 0.4	12.5 ± 0.3	7.3	3.6-58.0	6.0-9.0
CHO	16.4	17.4	49.8	37.6	26.6	31.9	42.2	55.6	38.5 - 56.1**	12-41.6	15-25
Ash	4.1 ± 0.0	2.4 ± 0.0	2.1 ± 0.0	1.8 ± 0.0	18.8 ± 0.2	14.5 ± 0.1	16.4 ± 0.0	8.8 ± 2.5	4.0-8.0	6.4	7.0-13.0

C. Mineral composition

The average mineral composition of ten different samples for nine minerals of dietary importance; including chloroplast rich fractions (CRFs), whole leaf materials and commercial algal preparations

	**Leaf CRFs (mg 100 g^{-1})**[1]				**Whole Leaf Materials (mg 100 g^{-1})** [1]				**Algal Preparations (mg 100 g^{-1})**	
	Spinach	Kale	Nettles	Grass	Spinach	Kale	Nettles	Grass	*Chlorella vulgaris* [6-8]	Spirulina (ssp. *Arthrospira*) [9,12]
Na	21 ± 5	39 ± 11	63 ± 4	44 ± 2	64 ± 11	9 ± 3	21 ± 11	32 ± 25	1350	250–900
Mg	337 ± 24	322 ± 1	396 ± 18	232 ± 28	398 ± 3	372 ± 11	737 ± 30	244 ± 31	340 - 440	195–400
P	298 ± 4	628 ± 11	370 ± 54	436 ± 9	87 ± 4	756 ± 6	413 ± 47	613 ± 2	960 - 1760	118–1000
K	1196 ± 68	518 ± 95	872 ± 64	1195 ± 234	12949 ± 403	4169 ± 95	2947 ± 73	4383 ± 417	50 - 2150	1360–1600
Ca	677 ± 15	920 ± 30	1736 ± 11	354 ± 4	2364 ± 0	2601 ± 163	6249 ± 188	323 ± 90	160 - 590	120–1500
Mn	7.7 ± 0.4	6.5 ± 0.7	7.9 ± 0.4	12.0 ± 3.4	5.1 ± 0.1	2.5 ± 0.0	14.1 ± 0.3	32.8 ± 2.9	400	1.9–5
Fe	31.4 ± 3.1	25.7 ± 9.1	42.1 ± 12	37.0 ± 0.6	16.0 ± 0.4	12.2 ± 0.1	21.1 ± 0.5	12.1 ± 3.1	200 - 680	28.5–170
Cu	2.0 ± 0.1	0.5 ± 0.1	1.0 ± 0.1	1.1 ± 0.4	1.7 ± 0.0	0.5 ± 0.0	1.0 ± 0.0	1.2 ± 0.1	190	1.2-6.1
Zn	3.60 ± 0.5	3.60 ± 0.2	3.60 ± 0.2	4.00 ± 0.7	14.90 ± 0.6	2.40 ± 0.0	3.10 ± 0.2	3.80 ± 0.5	550	2–7

D. Vitamin composition

The average vitamin composition of ten different samples for four key vitamins; including chloroplast-rich fractions (CRFs), whole leaf materials and commercial algal preparations

	**Leaf CRFs (mg 100 g^{-1})** [1]				**Whole Leaf Materials (mg 100 g^{-1})**[1]				**Algal Preparations (mg 100 g-1)**	
	Spinach	Kale	Nettles	Grass	Spinach	Kale	Nettles	Grass	*Chlorella vulgaris* [11]	Spirulina (ssp. *Arthrospira*)[9,12]
Provitamin A (i.e. beta carotene)	336 ± 10	247 ± 10	330 ± 0	255 ± 10	85 ± 10	86 ± 10	97 ± 10	75 ± 0	25 - 500^^	177 - 580
E (tocopherol)	62 ± 0	22 ± 0	51 ± 0	12 ± 0	10 ± 0	10 ± 0	14 ± 0.0	6 ± 0.0	3.0 - 45	5 - 100
K (phylloquinone)	-	-	-	-	-	-	-	-	0.3 - 4	0.0022 - 0.0286
C (ascorbic acid)	81 ± 20	112 ± 20	112 ± 30	112 ± 22	250-710 [1,10]	439 ± 42	214 ± 37	684 ± 36	7 - 1500	0.9 - 11.3

^As measured after homogenised, filtered and freeze dried
**As measured after freeze drying and then grinding with mortar and pestle*
*** Carbohydrates + Total Fibre Content*
^^ total carotenoids

References

1. Gedi MA, Briars R, Yuseli F, Zainol N, Darwish R, Salter AM, Gray DA. Component analysis of nutritionally rich chloroplasts: recovery from conventional and unconventional green plant species. Journal of Food Science and Technology. 2017 Aug;54(9):2746-57.

2. Kika J, Jakubczyk K, Ligenza A, Maciejewska-Markiewicz D, Szymczykowska K, Janda-Milczarek K. Matcha green tea: Chemical composition, phenolic acids, caffeine and fatty acid profile. Foods. 2024 Apr 11;13(8):116

3. Koláčková T, Sumczynski D, Minařík A, Yalçin E, Orsavová J. The effect of in vitro digestion on matcha tea (Camellia sinensis) active components and antioxidant activity. Antioxidants. 2022 Apr 30;11(5):889.

4. Saito A, Okada E, Matsumoto M, Takimoto H. Impact of updated standard tables of food composition on nutrient intakes in Japan. Journal of Food Composition and Analysis. 2019 Jun 1;79:5-11.

5. Fukuda K, Tategai K, Aoki M, Otsuki H, Aoi W. Matcha Green Tea Promotes Training-induced Anabolic Response of Skeletal Muscle in Mice. Journal of Food Research. 2026;15(1).

6. Jui TJ, Tasnim A, Islam SR, Manjur OH, Hossain MS, Tasnim N, Karmakar D, Hasan MR, Karim MR. Optimal growth conditions to enhance Chlorella vulgaris biomass production in indoor phyto tank and quality assessment of feed and culture stock. Heliyon. 2024 Jun 15;10(11).

7. Heo Y, Kim MY, Cho JY. Chlorella vulgaris, a representative edible algae as integrative and alternative medicine. Integrative Medicine Research. 2025 Aug 17:101228.

8. Safi C, Zebib B, Merah O, Pontalier PY, Vaca-Garcia C. Morphology, composition, production, processing and applications of Chlorella vulgaris: A review. Renewable and sustainable energy reviews. 2014 Jul 1;35:265-78.

9. Podgórska-Kryszczuk I. Spirulina—An invaluable source of macro-and micronutrients with broad biological activity and application potential. Molecules. 2024 Nov 15;29(22):5387.

10. Bergquist SÅ, Gertsson UE, Olsson ME. Influence of growth stage and postharvest storage on ascorbic acid and carotenoid content and visual quality of baby spinach (Spinacia oleracea L.). Journal of the Science of Food and Agriculture. 2006 Feb;86(3):346-55.

11. Bito T, Okumura E, Fujishima M, Watanabe F. Potential of Chlorella as a dietary supplement to promote human health. Nutrients. 2020 Sep;12(9):2524.

12. Agricultural Research Service (ARS), FoodData Central, Seaweed, spirulina, dried - https://fdc.nal.usda.gov/food-details/170495/nutrients. Visited February 2026.
